# DDR1 Drives Malignant Progression of Gastric Cancer by Suppressing HIF‐1α Ubiquitination and Degradation

**DOI:** 10.1002/advs.202308395

**Published:** 2024-07-18

**Authors:** Zhewei Wei, Jin Li, Li Zhong, Dongjie Yang, Wuguo Li, Wei Chen, Hao Zhou, Yulong He, Wu Song, Boyan Wang, Leli Zeng

**Affiliations:** ^1^ Department of Gastrointestinal Surgery The First Affiliated Hospital of Sun Yat‐sen University 58 Zhongshan 2nd Road Guangzhou Guangdong 510080 China; ^2^ Digestive Diseases Center, Guangdong Provincial Key Laboratory of Digestive Cancer Research Scientific Research Center The Biobank The Seventh Affiliated Hospital of Sun Yat‐Sen University No. 628 Zhenyuan Road Shenzhen Guangdong 518107 China; ^3^ Laboratory Animal Center The First Affiliated Hospital Sun Yat‐sen University 58 Zhongshan 2nd Road Guangzhou 510080 China; ^4^ Reproductive Medicine Center The First Affiliated Hospital of Sun Yat‐sen University 58 Zhongshan 2nd Road Guangzhou Guangdong 510080 China

**Keywords:** DDR1, ECM‐cells interaction, organoids, PDX, ubiquitination

## Abstract

The extracellular matrix (ECM) has been demonstrated to be dysregulated and crucial for malignant progression in gastric cancer (GC), but the mechanism is not well understood. Here, that discoidin domain receptor 1 (DDR1), a principal ECM receptor, is recognized as a key driver of GC progression is reported. Mechanistically, DDR1 directly interacts with the PAS domain of hypoxia‐inducible factor‐1α (HIF‐1α), suppresses its ubiquitination and subsequently strengthens its transcriptional regulation of angiogenesis. Additionally, DDR1 upregulation in GC cells promotes actin cytoskeleton reorganization by activating HIF‐1α/ Ras Homolog Family Member A (RhoA)/Rho‐associated protein kinase 1 (ROCK1) signaling, which in turn enhances the metastatic capacity. Pharmacological inhibition of DDR1 suppresses GC progression and angiogenesis in patient‐derived xenograft (PDX) and organoid models. Taken together, this work first indicates the effects of the DDR1‐HIF‐1α axis on GC progression and reveals the related mechanisms, providing experimental evidence for DDR1 as a therapeutic target for GC.

## Graphical abstract

This article demonstrates that DDR1, a primary member of extracellular matrix (ECM) receptors, is the key driver for gastric cancer (GC) progression, providing new insights into ECM‐cells interaction in tumor progression. The intracellular signaling pathway of DDR1 binds with HIF‐1α, suppressing the ubiquitin‐mediated degradation of HIF‐1α. Pharmacological inhibition of DDR1 effectively suppresses GC malignancy in patient‐derived xenograft (PDX) and organoid models.

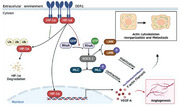

## 1 Introduction

Gastric cancer (GC) is the second leading cause of cancer‐related deaths worldwide, causing 769 000 deaths.^[^ ^1^ ^]^ GC can be curatively managed by surgical resection at early stages, with a 5‐year survival rate of more than 90%.^[^ ^2^, ^3^ ^]^ However, the molecular and clinical characteristics of GC are highly heterogeneous, leading to the poor prognosis of late‐stage GC. After spread to distant organs, the survival rate of patients with GC is only <10%.^[^ ^4^ ^]^ Recently, targeted therapy, which targets specific genes or proteins with improved efficacy and largely reduces the side effects, has emerged as a promising therapeutic approach for malignancies.^[^ ^5^ ^]^ However, the lack of specific treatment targets for GC limits the application of targeted therapy.^[^ ^6^ ^]^ Thus, it is highly urgent to explore new treatment targets for GC.

The extracellular matrix (ECM), an intricate network of extracellular macromolecules and minerals, is crucial for physical tissue maintenance as well as diverse cellular processes, including proliferation, adhesion, migration, polarity, differentiation, and apoptosis.^[^ ^7^ ^]^ Compelling evidence suggests that collagen, one of the most important components of the ECM,^[^ ^8^ ^]^ can regulate almost every biological characteristic of both tumor cells and the stroma predominantly through ECM‐cells interactions.^[^ ^9^ ^]^ Discoidin domain receptor 1 (DDR1) is a member of the transmembrane receptor tyrosine kinase (RTK) family and plays vital roles in ECM‐cells interactions as a collagen receptor.^[^ ^10^, ^11^ ^]^ Mechanistically, DDR1 mediates collagen‐induced signal transduction^[^ ^10^ ^]^ and triggers chemoresistance and immune evasion in tumors.^[^ ^12^, ^13^, ^14^, ^15^ ^]^ In GC, elevated DDR1 expression is correlated with worse survival,^[^ ^16^ ^]^ and inhibition of DDR1 is found to retard metastasis, indicating the crucial roles of DDR1 in GC.^[^ ^16^, ^17^ ^]^ However, the in‐depth mechanism underlying the role of DDR1 in GC remains largely unknown.

During malignant progression, angiogenesis is a crucial process facilitating ecosystem maintenance and tumor metastasis.^[^ ^18^ ^]^ The ECM plays critical roles in angiogenesis by secreting proangiogenic growth factors and stabilizing vascular tissues.^[^ ^19^ ^]^ Hypoxia‐inducible factor‐1α (HIF‐1α), a transcription factor, promotes the transcription of proangiogenic genes, such as vascular endothelial growth factor (VEGF) and vascular endothelial growth factor receptors (VEGFR). In addition to angiogenesis, many other cellular processes in tumors, including metastasis, aerobic glycolysis and inflammation, also depend on HIF‐1α.^[^ ^20^ ^]^ To date, the effects of DDR1, as a major mediator of ECM signaling, on angiogenesis and HIF‐1α are not well investigated.

Here, we revealed the mechanism by which DDR1 binds to HIF‐1α to inhibit its ubiquitin‐mediated degradation and promote angiogenesis in GC. In addition, our study suggested that HIF‐1α contributes to the activation of Ras Homolog Family Member A (RhoA)/Rho‐associated protein kinase 1 (ROCK1) signaling induced by DDR1 and consequently results in cytoskeleton reorganization and GC metastasis. Furthermore, our data showed that DDR1 inhibitors suppressed progression and angiogenesis in patient‐derived xenograft (PDX) and organoid models, highlighting the translational value of targeting the DDR1‐HIF1α axis for treating GC. Taken together, our studies first elucidated the intracellular mechanism of DDR1 in GC and its great therapeutic potential in translational medicine.

## 2 Results

### 2.1 High DDR1 Expression Positively Correlates with Malignant Progression in Human GC

**Figure 1:**
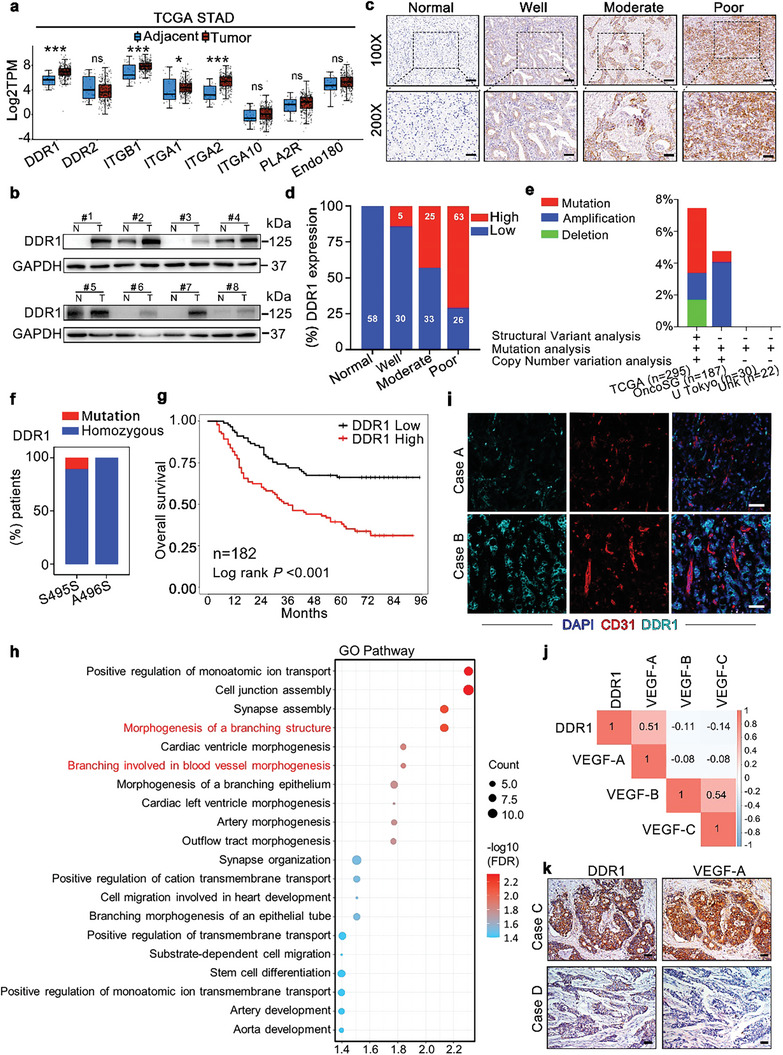
High DDR1 expression positively correlates with malignant progression in GC. a) Comparative gene expression analysis of collagen receptors (DDR1, DDR2, integrin β1, integrin α1, integrin α2, integrin α10, PLA2R, Endo180) between GC and adjacent normal tissues based on data from the Cancer Genome Atlas Stomach Adenocarcinoma (TCGA‐STAD) database (Cancer Genome Atlas Research Network, 2008). b) The DDR1 protein levels in GC tissues were higher than those in the matched stomach tissues, as measured by western blot analysis. The 8 paired tumor tissues and normal stomach tissues were from the FAHS cohort. c,d) DDR1 expression was significantly correlated with the aggressiveness of GC, as determined by IHC staining in normal gastric mucosa tissues (n = 58) and GC tissues (n = 182) from the FAHS cohort. Representative IHC staining images c) and quantification d) of DDR1 expression in normal gastric mucosa tissues (Normal) and in well, moderately and poorly differentiated GC tissues. Scale bar of upper panel: 100 µm. Scale bar of lower panel: 50 µm. e) DDR1 alterations in GC from the indicated studies are summarized based on data from cBioPortal. f) The S495S (13/131) and A495S (0/131) mutations of DDR1 were detected by sequencing based on a GC cohort from the FAHS (n = 131). g) GC patients with high DDR1 expression showed worse overall survival. Kaplan‒Meier analysis was performed according to DDR1 expression evaluated by IHC staining (log rank test; n = 182). h) Gene Ontology enrichment analysis of biological processes for up‐ and down‐regulated genes between HGC27 cells transfected with DDR1‐expressing versus control vector. i) Representative images of DDR1 (green) and CD31 (red) co‐staining in GC tissues, as revealed by IF staining (n = 32). Scale bar: 50 µm. j) Correlation matrix showing the correlations between DDR1 expression and VEGF‐A, VEGF‐B, and VEGF‐C expression in GC tissues based on data from the TCGA‐STAD cohort (Spearman test). k) Representative images of IHC staining for DDR1 and VEGF‐A in GC tissues. Scale bar: 25 µm. All data are presented as the mean ± SEM from three independent experiments. The P values in panels (a) were calculated by Student's *t‐*test. *P < 0.05, ***P < 0.001, ns: not significant.

The ECM is the major component of the tumor microenvironment (TME), exerting great influence on cellular phenotypes and signal transduction via ECM‐cell interactions.^[^ ^21^ ^]^ As collagen is the most abundant component of the ECM, collagen receptors in tumor cells have been proven to greatly affect the characteristics of tumors. In our work, we analyzed the levels of collagen receptors in GC and adjacent normal tissues, based on data from The Cancer Genome Atlas Stomach Adenocarcinoma (TCGA‐STAD) database. The results revealed that DDR1 was the only upregulated collagen‐binding receptor with kinase activity in GC (**Figure** **1**a). Data from GSE51575 and GSE13861 also confirmed the elevated expression of DDR1 in GC tissues (Figure S1a, Supporting Information). In addition, 8 paired nontumor and tumor tissues from GC patients in the First Affiliated Hospital of Sun Yat‐sen University (FAHS) were analyzed, and we found that DDR1 expression was also higher in the tumor tissues in this cohort (Figure 1b; Figure S1b, Supporting Information). The immunohistochemistry (IHC) results showed that DDR1 expression positively correlated with tumor grade in GC (Figure 1c,d), indicating the vital role of DDR1 in GC progression. Since DDR1 mutation has also been demonstrated to be positively correlated with poor prognosis in breast cancer,^[^ ^22^ ^]^ we analyzed DDR1 mutations based on cBioPortal and a GC cohort from our center.^[^ ^23^, ^24^ ^]^ However, only 14 mutations were found in 534 patients in cBioPortal (Figure 1e), and we found 13 S495S mutations, suggesting that mutation might not be the primary contributor to GC progression (Figure 1f). Finally, the high DDR1 group showed an inferior prognosis compared to the low DDR1 group (Figure 1g; Figure S1c, Supporting Information). Multivariate survival analyses suggested that DDR1 expression was a significant prognostic factor, indicating the critical role of DDR1 in GC (Tables S1–S3, Supporting Information).

However, the biological significance of DDR1 in GC remains largely unclear. Thus, we performed whole transcriptome analysis to investigate the biological function of DDR1. GO enrichment analysis revealed that blood vessel morphogenesis and morphogenesis of branching structures were significantly enriched in DDR1‐overexpressed GC cells (Figure 1h). During IHC staining, we noticed that the blood perfusion in the high DDR1 group seemed to be better than that in the low DDR1 group, indicating the proangiogenic effect of DDR1. Immunofluorescence (IF) staining of CD31, a marker of blood vessels, also revealed positive correlations between the DDR1 and CD31 levels (Figure 1i; Figure S1d, Supporting Information). To understand the mechanism underlying the angiogenic effect of DDR1, we analyzed the correlations between the expression of DDR1 and VEGFs (VEGF‐A, VEGF‐B and VEGF‐C), which are crucial regulators of angiogenesis, and a significant correlation between DDR1 and VEGF‐A expression was found (Figure 1j; Figure S1e, Supporting Information). Moreover, we confirmed that DDR1 expression was positively correlated with VEGF‐A expression by IHC (Figure 1k; Figure S1f, Supporting Information), further indicating that VEGF‐A might be one of the main mediators of the proangiogenic effects of DDR1.

### 2.2 DDR1 Induces Angiogenesis through HIF‐1α in GC Cells

**Figure 2:**
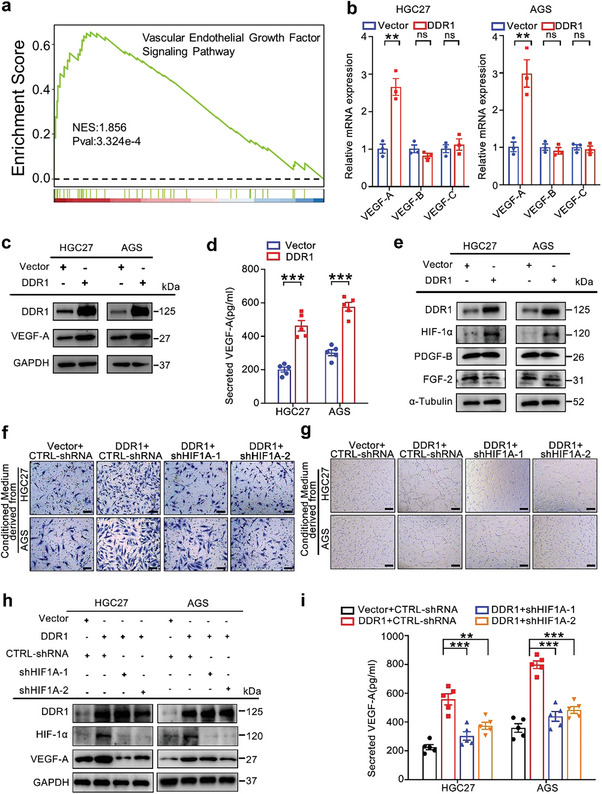
DDR1 induces angiogenesis through HIF‐1α in GC cells. a) GSEA of Vascular Endothelial Growth Factor Signaling in HGC27 cells with DDR1‐overexpressing vector control vector. b) DDR1 overexpression upregulated *VEGF‐A* expression in GC cells. Real‐time PCR analysis was performed to measure the mRNA levels of *VEGF‐A, VEGF‐B*, and *VEGF‐C*. c) Ectopic DDR1 expression increased the VEGF‐A protein level in GC cells. The VEGF‐A protein level was measured by western blot analysis in DDR1‐overexpressing and control GC cells. d) DDR1 increased the VEGF‐A level in CM from GC cells. The level of secreted VEGF‐A protein in CM derived from HGC27 or AGS cells stably expressing DDR1, or control vector was evaluated by ELISA. e) Western‐blot analysis was performed to evaluate the expression of VEGF‐A regulators, including HIF‐1α, PDGF and FGF2. f,g) Silencing of *HIF1A* in DDR1‐overexpressing GC cells inhibited the migration f) and tube formation g) of HUVECs. HUVECs were incubated with CM from HGC27 or AGS cells transduced with the indicated vectors. h) Knockdown of *HIF1A* suppressed VEGF‐A expression in DDR1‐overexpressing GC cells. Western blot analysis was performed to measure the protein level of VEGF‐A in HGC27 or AGS cells transfected with the indicated vectors. i) Silencing of *HIF1A* inhibited VEGF‐A secretion into the CM from DDR1‐overexpressing GC cells. ELISA was performed to measure the level of secreted VEGF‐A in the CM from GC cells transfected with the indicated vectors. All data are presented as the mean ± SEM from three independent experiments. The *P* values in panels (b) and (d) were calculated by Student's *t*‐test. The *P* values in panels (i) were calculated by one‐way ANOVA. **P < 0.01, ***P < 0.001, ns: not significant.

As a hallmark of cancer, angiogenesis is indispensable for tumor growth and metastasis.^[^ ^18^ ^]^ To further elucidate the influence of DDR1 on angiogenesis in vitro, HGC27 and AGS cells with ectopic DDR1 expression were constructed (Figure S2a,b, Supporting Information). Endothelial cell recruitment and tube formation assays were conducted to explore the impact of DDR1 on endothelial cells in vitro. Treatment with conditioned medium from DDR1‐overexpressing GC cells significantly promoted the migration of human umbilical vein endothelial cells (HUVECs) (Figure S2c,d, Supporting Information) and obviously increased the formation of capillary‐like structures and branches (Figure S2e,f, Supporting Information). Meanwhile, *DDR1* knockout (KO) in MKN74 cells suppressed the migration of HUVECs and the formation of capillary‐like structures and branches (Figure S2g,h, Supporting Information). To further explore the mechanism of the proangiogenic effect of DDR1, we performed GSEA analysis and showed that the Vascular Endothelial Growth Factor Signaling Pathway was significantly upregulated in DDR1‐overexpressed HGC27 cells (**Figure** **2**a). We then sought to elucidate the influence of DDR1 on VEGFs expression in GC cell lines. RT‐PCR analyses showed that DDR1 overexpression increased the mRNA level of *VEGF‐A* but not *VEGF‐B* or *VEGF‐C* (Figure 2b). The western blot and ELISA results further confirmed the changes in VEGF‐A protein expression induced by DDR1 (Figure 2c,d; Figure S2k,l, Supporting Information), indicating that DDR1 might promote capillary formation by increasing the VEGF‐A level.

Previous research has indicated that HIF‐1α, PDGF, and FGF2 are the primary regulators of VEGF‐A.^[^ ^25^ ^]^ The western blot showed that only HIF‐1α was upregulated in DDR1‐overexpressed GC cells, but not PDGF or FGF2 (Figure 2e). HIF‐1α is one of the major drivers of angiogenesis and shows crucial influence on VEGF‐A expression as a transcription factor.^[^ ^20^ ^]^ To investigate whether HIF‐1α is involved in DDR1‐induced angiogenesis, we silenced *HIF1A* and found that the migration and tube formation of HUVECs caused by treatment with conditioned medium from GC cells overexpressing DDR1 were dramatically suppressed (Figure 2f,g; Figure S2n,o, Supporting Information). RT‐PCR analysis revealed that *HIF1A* knockdown significantly decreased the mRNA levels of *VEGF‐A* in cells with ectopic DDR1 expression (Figure S2p, Supporting Information), and this decrease was further verified by western blot and ELISA (Figure 2h,i). Furthermore, we intended to investigate the effect of *HIF1A* restoration in *DDR1* KO GC cells. Endothelial cell recruitment and tube formation assays showed that *HIF1A* overexpression in *DDR1* KO GC cells rescued the migration and capillary tube formation of HUVECs (Figure S3a,b, Supporting Information). Westen blot analyses suggested that *HIF1A* overexpression induced VEGF‐A expression in *DDR1* KO GC cells (Figure S3c, Supporting Information). These data together suggested that DDR1‐induced angiogenesis in GC is mediated by HIF‐1α.

### 2.3 DDR1 Stabilizes the HIF‐1α Protein by Suppressing its Ubiquitination

**Figure 3:**
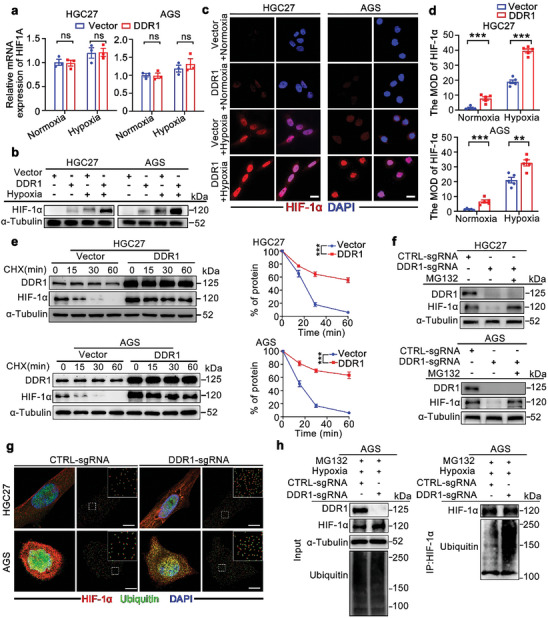
DDR1 stabilizes the HIF‐1α protein by suppressing its ubiquitination. a) DDR1 overexpression did not change the mRNA level of *HIF1A* in HGC27 and AGS cells under normoxic or hypoxic conditions. b) DDR1 overexpression upregulated HIF‐1α protein expression in GC cells. Western blot analysis was performed to measure the HIF‐1α protein level in HGC27 and AGS cells transfected with the indicated vectors and cultured under normoxic or hypoxic conditions for 24 h. c,d) Representative IF images c) and quantification d) of HIF‐1α expression in HGC27 and AGS cells transfected with the DDR1‐expressing or control vector and cultured under normoxic or hypoxic conditions. Nuclei were stained with DAPI. Scale bar: 20 µm. e) DDR1 overexpression inhibited HIF‐1α degradation in GC cells. HGC27 and AGS cells were treated with 10 µg mL^−1^ cycloheximide (CHX) for the indicated times, and the HIF‐1α level was measured by western blot analysis. f) Treatment with the proteasome inhibitor MG132 suppressed the HIF‐1α protein degradation induced by *DDR1 KO* in GC cells. MG132 (10 µM) was used to treat GC cells transfected with the indicated vectors. g) *DDR1* knockout increased the colocalization of ubiquitin and HIF‐1α in GC cells. Representative images of the colocalization of ubiquitin (green) and HIF‐1α (red), as determined by IF analysis. Scale bar: 10 µm. h) Silencing of DDR1 increased the ubiquitination level of HIF‐1α in AGS cells. MG132 (10 µM) was used to treat AGS cells transfected with Control sgRNA and DDR1‐sgRNA. All data are presented as the mean ± SEM of three independent experiments. The *P* values in panels (a), (d), (g) were calculated by Student's *t*‐test. The *P* values in panels (e) were calculated by two‐way ANOVA. **P < 0.01, ***P < 0.001, ns: not significant.

We then aimed to elucidate the influence of DDR1 on HIF‐1α expression in GC cells. Western blot analyses suggested that DDR1 overexpression increased HIF‐1α protein levels under both normoxic and hypoxic conditions, while the mRNA level of *HIF1A* remained unchanged (**Figure** **3**a,b). Consistent with this finding, IF analysis showed that HIF‐1α was upregulated by DDR1 overexpression in GC cells (Figure 3c,d). These data suggested that the increase in the HIF‐1α protein level might be attributed to posttranslational regulation in DDR1‐overexpressing GC cells.

The ubiquitin‐proteasome system is a main mechanism contributing to HIF‐1α degradation in tumors.^[^ ^26^ ^]^ To investigate whether DDR1 modulates HIF‐1α by influencing its protein stability, DDR1‐overexpressing GC cells were treated with the protein synthesis inhibitor cycloheximide (CHX). The results showed that DDR1 overexpression significantly attenuated the degradation of HIF‐1α protein in GC cells (Figure 3e). To determine whether ubiquitin‐proteasome‐mediated degradation was responsible for the increase in the HIF‐1α protein level caused by DDR1, the proteasome inhibitor MG132 was utilized in *DDR1* KO GC cells. The results showed that the suppression of ubiquitin‐proteasome‐mediated degradation by MG132 reversed the decrease in the HIF‐1α level in *DDR1* KO GC cells (Figure 3f). IF staining and western blot analyses both suggested that GC cells with *DDR1* KO exhibited increased ubiquitination of HIF‐1α (Figure 3g,h). In summary, these data suggested that DDR1 stabilizes the HIF‐1α protein by suppressing its ubiquitination.

### 2.4 DDR1 Directly Interacts with the PAS Domain of HIF‐1α

**Figure 4:**
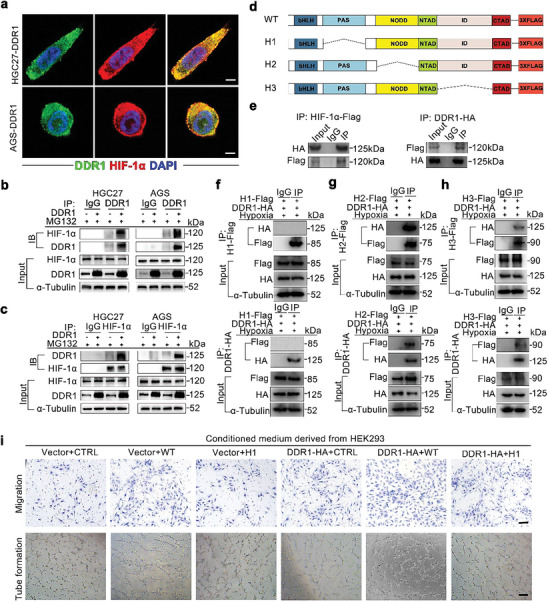
DDR1 directly interacts with the PAS domain of HIF‐1α. a) IF staining showed that DDR1 (green) and HIF‐1α (red) were colocalized in DDR1‐overexpressing GC cells. Scale bar: 10 µm. b,c) DDR1 interacted with HIF‐1α in GC cells. HGC27 and AGS cells transfected with the DDR1‐expressing or control vector were utilized for co‐IP assays using the anti‐DDR1 (b) or anti‐HIF‐1α c) antibody. d) Schematic representation of full‐length and different flag‐tagged deletion mutants of HIF‐1α. e) The lysates of HEK293T cells transfected with the indicated vectors were subjected to co‐IP with the anti‐HA (left) or anti‐Flag (right) antibody, and the precipitates were analysed by western blotting. f,g,h) The PAS domain was indispensable for the interaction of HIF‐1α with DDR1. HEK293T cells were transfected with vectors expressing the H1‐Flag f), H2‐Flag g) or H3‐Flag h) deletion mutants of HIF‐1α together with the DDR1‐HA vector for co‐IP assays. i) The PAS domain mediated the DDR1‐induced migration and tube formation of HUVECs. Representative images of migration (upper panel) and tube formation (lower panel) of HUVECs cultured with CM derived from cells co‐transfected with the indicated vectors. Scale bar for migration images (upper panel): 100 µm. Scale bar for tube formation images (lower panel): 200 µm.

To clarify the mechanism by which DDR1 regulates ubiquitin‐proteasome‐mediated degradation of HIF‐1α, IF staining was performed to investigate the intracellular distributions of DDR1 and HIF‐1α in AGS and HGC27 cells. Interestingly, marked colocalization of DDR1 and HIF‐1α was observed which demonstrates the interaction between DDR1 and HIF‐1α in situ (**Figure** **4**a). Then, we performed co‐IP assays to further confirm the interactions between DDR1 and HIF‐1α. The results showed that HIF‐1α and DDR1 coprecipitated with each other, further revealing that DDR1 binds with HIF‐1α in GC cell (Figure 4b,c).

To explore the domain of HIF‐1α involved in its binding to DDR1, plasmids expressing flag‐tagged HIF‐1α core domain deletion mutants were constructed and transfected into HEK293T cells (Figure 4d,e). The Per/ARNT/Sim (PAS) domain of HIF‐1α was the key domain in its interaction with DDR1 (Figure 4f–h). Furthermore, the PAS domain of HIF‐1α was also indispensable for mediating DDR1‐induced VEGF‐A upregulation as well as HUVEC migration and tube formation (Figure 4i; Figure S4a–c, Supporting Information). Correlation analysis based on TCGA‐STAD data revealed strong correlations between the expression of DDR1 and HIF‐1α target genes, including *GLUT1*, *ALDOA*, *PGK1* and *TFRC*, as further confirmed in AGS and HGC27 cell lines (Figure S4d,e, Supporting Information). Consistent with this finding, the correlation between DDR1 and HIF‐1α expression was also detected by IHC in 182 GC tissues (Figure S4f,g, Supporting Information). These data suggested that the PAS domain of HIF‐1α is responsible for its direct interaction with DDR1 and is vital for DDR1‐induced angiogenesis.

### 2.5 DDR1 Regulates Actin Cytoskeleton Reorganization via RhoA/ROCK1 Signaling

**Figure 5:**
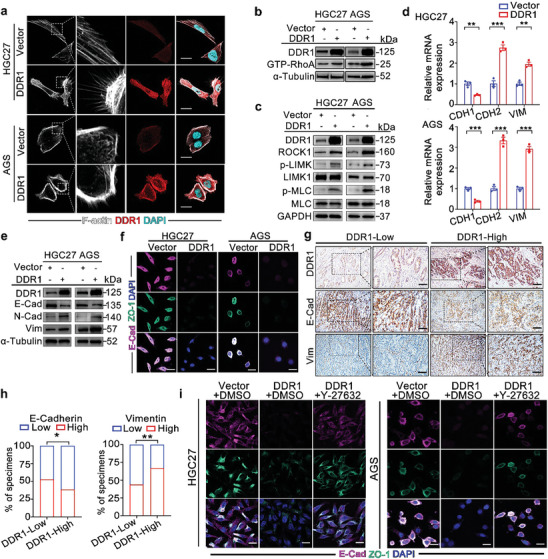
DDR1 regulates actin cytoskeleton reorganization via RhoA/ROCK1 signaling in GC cells. a) DDR1 overexpression triggered actin cytoskeleton reorganization in GC cells. IF analysis of F‐actin (white) using phalloidin staining and DDR1 (red) in HGC27 and AGS cells transfected with the DDR1‐expressing or control vector. Scale bar: 20 µm. b) DDR1 significantly enhanced RhoA activity in GC cells. RhoA activity in HGC27 and AGS cells transfected with the DDR1‐expressing or control vector was determined by a GTP‐RhoA pulldown assay using GST‐Rhotekin‐RBD. c) DDR1 promoted ROCK1 expression and the phosphorylation of LIMK and MLC in GC cells. Western blot analysis was performed to measure ROCK1, p‐LIMK, LIMK1, p‐MLC and MLC levels in HGC27 and AGS cells transfected with the DDR1‐expressing and control vectors. d,e) DDR1 overexpression induced EMT in GC cells. E‐cadherin (*CDH1*), N‐cadherin (*CDH2*) and vimentin (*VIM*) expression were measured by RT‐PCR d) and western blotting e) in HGC27 and AGS cells transfected with the DDR1‐expressing or control vector. f) DDR1 suppressed E‐cadherin and ZO‐1 expression in GC cells. IF staining of E‐Cadherin (purple) and ZO‐1 (green) was performed in HGC27 and AGS cells transfected with the DDR1‐expressing or control vector. Nuclei were stained with DAPI. Scale bar: 25 µm. g,h) DDR1 expression was correlated with E‐Cadherin and Vimentin expression in primary human GC tissues. Representative IHC images g) and quantification h) of E‐Cadherin and Vimentin expression in DDR1‐high and DDR1‐low GC tissues based on the FAHS cohort (n = 182). Scale bar: 50 µm. i) ROCK1 inhibition attenuated the downregulation of E‐cadherin and ZO‐1 in DDR1‐overexpressing GC cells. Y‐27632 (10 µM) was used to treat HGC27 and AGS cells transfected with the indicated vectors. IF staining of E‐cadherin (purple) and ZO‐1 (green). Nuclei were stained with DAPI. Scale bar: 25 µm. All data are presented as the mean ± SEM from three independent experiments. The *P* values in panels (d) were calculated by Student's *t*‐test. The *P* values in panels (h) were calculated by χ^2^ test. *P < 0.05, **P < 0.01, ***P < 0.001.

As the impact of DDR1 on HUVECs was elucidated, we made further efforts to clarify its impacts on GC cells. Accumulating evidence implicates the actin cytoskeleton as a critical regulator of many cellular processes.^[^ ^27^ ^]^ Recent studies suggest that DDR1 exerts diverse effects on actin cytoskeleton reorganization in different cell types.^[^ ^28^, ^29^, ^30^ ^]^ Phalloidin staining was performed to visualize the actin cytoskeleton and showed that ectopic DDR1 expression in GC cells induced increased actin stress fiber formation. In addition, the formation of some specialized membrane structures, such as pseudopodia, was also enhanced by DDR1 overexpression (**Figure** **5**a; Figure S5a, Supporting Information), suggesting the increased migratory capacity of GC cell lines. RhoA, a small GTPase protein, is one of the main regulators of actin cytoskeleton reorganization.^[^ ^31^ ^]^ Activation of RhoA was found to induce actin cytoskeleton remodeling in vascular smooth muscle cells.^[^ ^30^ ^]^ However, the effects of DDR1 on RhoA activation have seldom been reported previously. Thus, we investigated whether DDR1 affected the reorganization of actin filaments by influencing RhoA activity in GC cells. The results showed that DDR1 overexpression significantly enhanced RhoA activity (Figure 5b). ROCK1 is the main downstream effector of RhoA, which subsequently phosphorylates LIM kinase (LIMK) and myosin light chain (MLC) to regulate actin filament organization.^[^ ^32^ ^]^ Western blot analyses suggested that the phosphorylation levels of LIMK and MLC were obviously higher in HGC27 and AGS cells overexpressing DDR1 (Figure 5c). Additionally, we observed that *DDR1* KO had a profound impact on RhoA/ROCK1 signaling in GC cells (Figure S5b,c, Supporting Information).

While actin cytoskeleton reorganization has profound influences on cell migration, morphogenesis and tumor metastasis,^[^ ^33^ ^]^ we intended to elucidate the effect of DDR1 on the metastatic capacity of GC cells. Transwell and scratch wound healing assays were conducted to investigate how DDR1 influences the migratory and invasive abilities of GC cells. We demonstrated that DDR1 overexpression enhanced the migratory and invasive abilities of GC cells (Figure S6a,b, Supporting Information). Epithelial‐to‐mesenchymal transition (EMT) is a crucial process in tumor metastasis defined by the conversion of epithelial tumor cells to cells with mesenchymal properties.^[^ ^34^ ^]^ Here, we demonstrated that the expression of E‐cadherin, an epithelial marker, was reduced in HGC27 and AGS cell lines overexpressing DDR1, while the expression of mesenchymal markers, including N‐cadherin and vimentin, was increased (Figure 5d,e). In contrast, elimination of DDR1 suppressed the EMT process in HGC27 and AGS cells, as shown by RT‐PCR analysis in Figure S5d (Supporting Information). IF analysis showed similar results (Figure 5f; Figure S6c, Supporting Information). Moreover, IHC analysis was conducted to explore the correlations between DDR1 and EMT markers in clinical GC samples. The results revealed that GC samples with high DDR1 expression showed upregulated Vimentin expression compared to those with low DDR1 expression (62/93 versus 39/89; *P* = 0.003). In addition, a negative correlation between DDR1 and E‐cadherin expression was observed in patient GC samples (Figure 5g,h). Next, we utilized the ROCK inhibitor Y‐27632 to explore whether ROCK signaling blockade would decrease the DDR1‐promoted metastatic ability. Y‐27632 was found to effectively suppress ROCK and its signaling pathway molecules that were induced in DDR1‐overexpressing GC cells (Figure S7a, Supporting Information) and to reverse the effect of DDR1 overexpression on EMT marker expression and metastatic capacity (Figure S7b–e, Supporting Information). Similarly, the IF results also suggested that Y‐27632 attenuated the DDR1‐induced changes in E‐cadherin and ZO‐1 expression (Figure 5i; Figure S7f, Supporting Information). These data revealed that DDR1 regulates actin cytoskeleton reorganization through RhoA/ROCK1 signaling in GC.

### 2.6 HIF‐1α is Required for the Actin Cytoskeleton Reorganization Induced by DDR1

**Figure 6:**
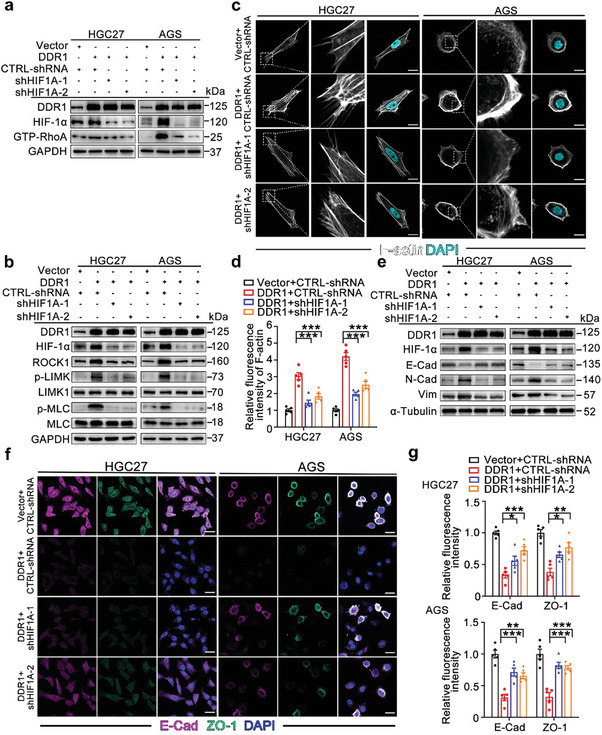
HIF‐1α is required for the actin cytoskeleton reorganization induced by DDR1. a) *HIF1A* silencing blocked RhoA activation triggered by DDR1 in GC cells. RhoA activity in HGC27 and AGS cells transduced with the indicated vectors was measured by a GTP‐RhoA pulldown assay using GST‐Rhotekin‐RBD. b) *HIF1A* knockdown suppressed DDR1‐induced expression of ROCK and phosphorylation of LIMK and MLC. Western blot analysis was performed to measure ROCK1, p‐LIMK, LIMK1, p‐MLC and MLC protein levels. c,d) Knockdown of *HIF1A* suppressed actin cytoskeleton reorganization in DDR1‐overexpressing GC cells. Representative images c) and quantification d) of F‐actin (white) by phalloidin staining with confocal fluorescence microscopy. Nuclei were stained with DAPI. Scale bar: 20 µm. e) Silencing of *HIF1A* inhibited DDR1‐induced EMT in GC cells. Western blot analysis was performed to evaluate N‐cadherin, E‐cadherin and Vimentin expression in HGC27 and AGS cells transduced with the indicated vectors. f,g) HIF‐1α mediated DDR1‐induced EMT in GC cells. The expression levels of E‐Cadherin (purple) and ZO‐1 (green) were assessed by IF staining. Scale bar: 25 µm. All data are presented as the means ± SEMs from three independent experiments. The *P* values in panels (d), (g) were calculated using one‐way ANOVA. *P < 0.05, **P < 0.01, ***P < 0.001.

Recent studies have indicated that HIF‐1α is involved in actin cytoskeleton reorganization in several kinds of cells and tissues.^[^ ^35^ ^]^ Hence, we speculated that HIF‐1α might also contribute to the actin cytoskeleton reorganization regulated by DDR1. Since RhoA/ROCK1 signaling has been reported to correlate with the actin cytoskeleton reorganization caused by DDR1, we tried to show whether the effect of DDR1 on actin cytoskeleton reorganization is dependent on HIF‐1α/RhoA/ROCK1 signaling in GC cells. In DDR1‐overexpressing GC cell lines, silencing *HIF1A* significantly suppressed the activity of RhoA and its downstream effectors, including phosphorylated LIMK and phosphorylated MLC (**Figure** **6**a,b). Furthermore, phalloidin staining revealed that the formation of actin stress fibers and pseudopodia was decreased after *HIF1A* knockdown (Figure 6c,d). Because RhoA/ROCK1 signaling is also closely related to tumor EMT and metastasis,^[^ ^36^ ^]^ we further investigated the role of HIF‐1α in DDR1‐induced EMT and metastasis in GC. The results showed that *HIF1A* knockdown could retard the EMT caused by DDR1 overexpression in GC cells (Figure 6e–g; Figure S8a,b, Supporting Information). Similarly, *HIF1A* knockdown also reduced the metastatic ability of GC cells with DDR1 overexpression (Figure S8c–e, Supporting Information). In summary, these results demonstrated that HIF‐1α contributed to actin cytoskeleton reorganization by mediating the activation of RhoA/ROCK1 signaling in GC cells.

### 2.7 DDR1 Promotes GC Progression via HIF‐1α In Vivo

**Figure 7:**
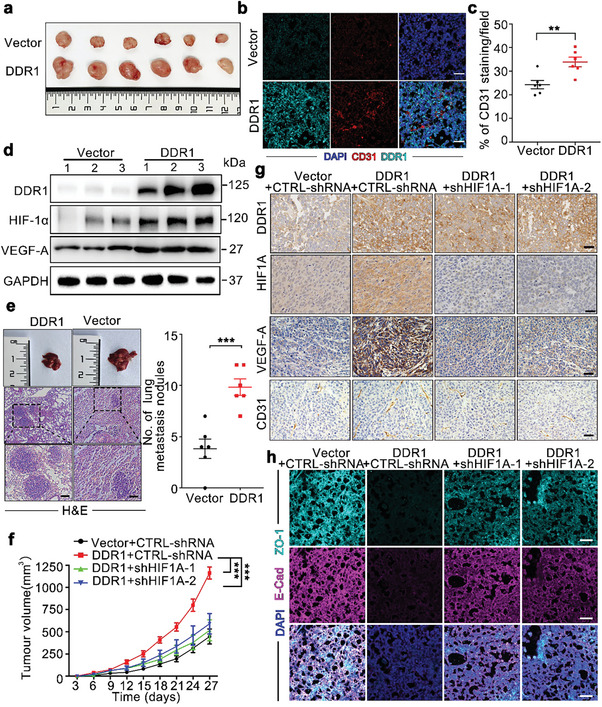
DDR1 promotes GC progression via HIF‐1α in vivo. a) Representative images of subcutaneous tumors established by AGS‐DDR1 and AGS‐Vector cells. b,c) DDR1 overexpression increased the microvessel density in subcutaneous tumors. Representative micrographs b) and quantification c) of CD31 (red) and DDR1 (green) expression in xenograft tumors from nude mice via IF staining. Nuclei were stained with DAPI. Scale bar: 50 µm. d) DDR1 overexpression promoted HIF‐1α and VEGF‐A expression in vivo. Western blot analysis was performed to measure DDR1, HIF‐1α and VEGF‐A protein levels in the subcutaneous tumors from the 2 groups. e) DDR1 overexpression significantly increased the number of lung metastatic nodules in mouse models. Representative images (left) and quantification (right) of lung metastases established by tail vein injection, as determined by H&E staining. Scale bar: 25 µm. f) *HIF1A* silencing suppressed DDR1‐induced tumor growth. The growth of tumors formed by AGS‐DDR1 and AGS‐Vector cells was measured every three days (n = 6/group). g) *HIF1A* knockdown inhibited the VEGF‐A and CD31 expression induced by DDR1. Representative micrographs of IHC staining for DDR1, HIF‐1α, VEGF‐A and CD31 in subcutaneous tumors. Scale bar: 50 µm. h) *HIF1A* silencing attenuated DDR1‐induced EMT in vivo. Representative micrographs of IF staining for E‐cadherin (purple) and ZO‐1 (green) in tumors from nude mice. Nuclei were stained with DAPI. Scale bar: 50 µm. All data are presented as the means ± SEMs from three independent experiments. The *P* values in panels (c) and (e) were calculated using Student's *t*‐test. The *P* values in panels (f) were calculated using two‐way ANOVA. *P < 0.05, **P < 0.01, ***P < 0.001, ns, not significant.

To further explore the influence of DDR1 on tumor progression in vivo, mouse xenograft models were applied. AGS cells with and without DDR1 overexpression (referred to as AGS‐DDR1 and AGS‐Vector, respectively) were subcutaneously injected to establish the xenograft model (n = 6 mice per group). The tumors were harvested 4 weeks later (**Figure** **7**a). Tumors formed by AGS‐DDR1 cells exhibited an increased volume and weight (Figure S9a,b, Supporting Information). IF staining of CD31 suggested that tumors with DDR1 overexpression showed an increased microvessel density (Figure 7b,c). To clarify the role of DDR1 in tumor angiogenesis in vivo, HIF‐1α and VEGF‐A expression were measured in xenograft tumor samples. Western blot analysis suggested that the HIF‐1α and VEGF‐A levels were higher in AGS‐DDR1 tumor xenografts, consistent with the in vitro findings (Figure 7d). Furthermore, the western blot analyses showed that the RhoA signaling was also upregulated in AGS‐DDR1 tumor xenografts (Figure S9c,d, Supporting Information), and DDR1 overexpression promoted EMT process in tumor xenografts (Figure S9e, Supporting Information). To explore the impact of DDR1 on the metastatic capacity, a lung metastasis model was established by injecting GC cells into the tail vein. H&E staining showed that DDR1 overexpression increased the number of lung micrometastatic nodules (Figure 7e).

Furthermore, *HIF1A* expression was silenced to elucidate its functional role in DDR1‐induced angiogenesis and metastasis in vivo. Silencing of *HIF1A* significantly attenuated DDR1‐increased tumor growth in nude mice (Figure 7f; Figure S9f,g, Supporting Information). The CD31 and VEGF‐A levels in AGS‐DDR1 xenografts were also decreased after *HIF1A* knockdown (Figure 7g; Figure S9h,i, Supporting Information), suggesting that HIF‐1α is correlated with the in vivo angiogenic effect of DDR1. In addition, *HIF1A* silencing also reversed the EMT caused by DDR1 overexpression (Figure 7h; Figure S9j, Supporting Information), and lung metastasis of AGS‐DDR1 in vivo was inhibited by *HIF1A* knockdown (Figure S9k,l, Supporting Information). Taken together, these data indicated that HIF‐1α is the key mediator of DDR1‐induced angiogenesis and metastasis in GC.

### 2.8 Pharmacological Inhibition of DDR1 Suppresses Malignant Progression of GC in PDX and Organoid Models

**Figure 8:**
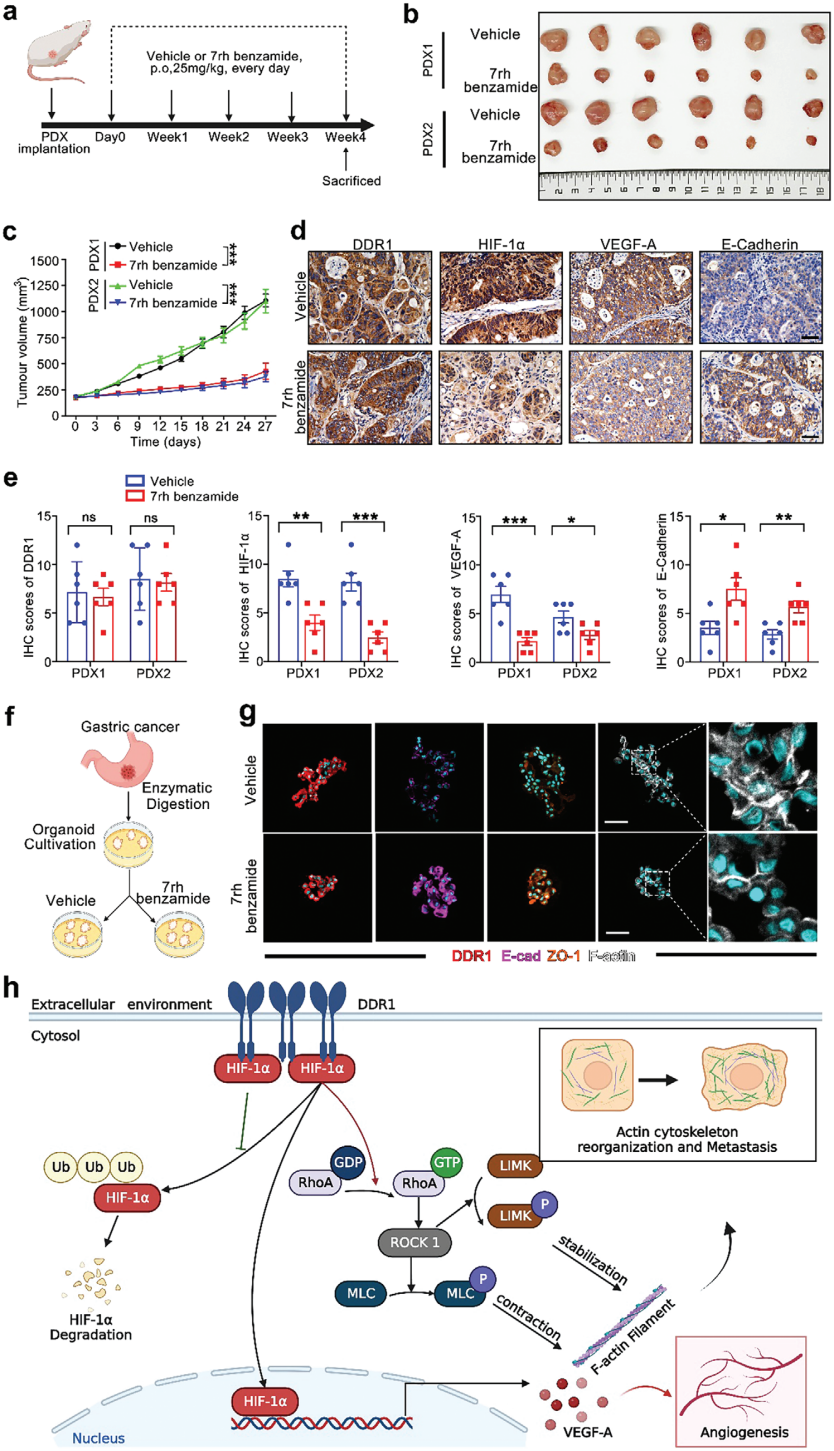
Pharmacological inhibition of DDR1 suppresses malignant progression in PDX and organoid models. a) Experimental protocol of the PDX models. b) Representative images of tumors harvested from the 2 PDX models. c) Pharmacological inhibition of DDR1 suppressed tumor growth in the PDX models. The DDR1 inhibitor 7rh benzamide was administered at a dose of 25 mg kg^−1^ by oral gavage every day. d,e) Treatment with 7rh benzamide decreased the expression of HIF‐1α, VEGF‐A, and E‐Cadherin in the PDX models. Representative images d) and quantification e) of DDR1, HIF‐1α, VEGF‐A, and E‐Cadherin expression determined by IHC staining in tumors from PDX models treated with 7rh benzamide or vehicle. Scale bar: 50 µm. f) Schematic illustration of organoid models derived from dissected human GC tissues. g) IF images of E‐Cadherin, ZO‐1, and F‐actin in GC organoids treated with 7rh benzamide or vehicle. Scale bar: 50 µm. h) Schematic illustration of the model that DDR1 interacts with HIF‐1α to suppress its degradation, and consequently promote malignant progression of GC. All data are presented as the means ± SEMs from three independent experiments. The *P* values in panels (c), (e) were calculated using Student's *t*‐test. *P < 0.05, **P < 0.01, ***P < 0.001, ns: not significant.

In recent years, PDX and organoid models, both of which directly originate from human tumor specimens, have emerged as promising approaches for translational research in cancer treatment.^[^ ^37^ ^]^ To investigate the therapeutic potential of DDR1 as a treatment target for GC, a highly selective inhibitor of DDR1, 7rh benzamide, was used to block DDR1 signaling in PDX and organoid models. In our study, PDX models (PDX1 and PDX2) were constructed with passage three tumors derived from two patients’ GC samples (Figure S10a, Supporting Information). When the xenograft tumors were palpable (≈200 mm^3^), mice with similar tumor volumes were randomly administered 7rh benzamide or vehicle for 4 weeks (**Figure** **8**a). Treatment with 7rh benzamide had no obvious influence on body weight (Figure S10b, Supporting Information) but effectively inhibited tumor growth in both the PDX1 and PDX2 models (Figure 8b,c; Figure S10c, Supporting Information). The IHC results demonstrated that DDR1 inhibition with 7rh benzamide blocked HIF‐1α and VEGF‐A expression and upregulated E‐cadherin expression in the PDX models (Figure 8d,e). Then, we established an organoid model from primary GC specimens to assess the efficacy of 7rh benzamide (Figure 8f). The results of IF staining demonstrated that in the organoid model, pharmacological inhibition of DDR1 suppressed cytoskeletal reorganization and simultaneously enhanced E‐cadherin and ZO‐1 expression (Figure 8g; Figure S10d, Supporting Information). Collectively, these results in PDX and organoid models elucidated the significant role of DDR1 in promoting GC malignancy and pointed out the potential of DDR1 as a therapeutic target for GC in translational medicine. In summary, our results suggest that DDR1 directly interacts with HIF‐1α to inhibit its ubiquitin‐mediated degradation, which in turn promotes the malignant progression of GC.

## 3 Discussion

Previous studies have well demonstrated the significant role of ECM‐cells interactions in regulating the TME,^[^ ^38^, ^39^ ^]^ while the precise mechanism by which the ECM affects tumor progression remains largely unclear. Interactions between collagen, a fundamental component of the ECM, and its receptors are a main mechanism by which the ECM exerts its influence on tumor progression.^[^ ^40^ ^]^ In the present study, we demonstrated that DDR1 was the only collagen‐binding receptor with kinase activity overexpressed in GC. Both in vivo and in vitro results suggested that DDR1 was involved in angiogenesis and metastasis, probably by inhibiting the ubiquitin‐mediated degradation of HIF‐1α and activating the HIF‐1α/RhoA/ROCK1 signaling pathway. By studies in PDX mouse and organoid models, as well as treatment with 7rh benzamide, our group further confirmed the potential of DDR1 as a novel therapeutic target in GC.

Emerging evidence reveals aberrant DDR1 expression in various kinds of malignancies, such as glioblastoma, lung adenocarcinoma, and head and neck squamous cell carcinoma.^[^ ^12^, ^14^, ^41^ ^]^ Some studies have demonstrated that somatic mutations may contribute to elevated DDR1 expression and correlate with patient survival in several types of cancers, including breast cancer^[^ ^22^ ^]^ and endometrial cancer.^[^ ^42^ ^]^ In contrast, our results demonstrated that mutations of DDR1 are relatively rare in GC based on the data from cBioPortal and a cohort from our medical center, indicating that mutation might not be the main factor contributing to DDR1 upregulation in GC.

Accumulating studies have suggested that GC exhibits a highly angiogenic phenotype, revealing angiogenesis as a promising target.^[^ ^18^, ^43^ ^]^ Recent evidence demonstrated that ECM components are critical to tumor angiogenesis, though the in‐depth mechanism were yet to unravel. Interestingly, our results showed a positive correlation between DDR1 expression and microvessel density in clinical GC samples. Further in vivo and in vitro experiments confirmed the proangiogenic effect of DDR1. Thus, our study provided evidence that DDR1 as a collagen receptor mediated ECM‐induced angiogenesis in GC.

Currently, the mechanism underlying DDR1's function in tumor angiogenesis has not been reported yet. HIF‐1α, PDGF, and FGF2 are the primary regulators of tumor angiogenesis.^[^ ^25^ ^]^ Our data showed that HIF‐1α was upregulated by DDR1, but not PDGF or FGF2. In addition, our results suggested that DDR1 expression was positively correlated with HIF‐1α expression in clinical GC samples. HIF‐1α functions as a transcription factor and exerts a strong influence on angiogenesis by regulating the expression of angiogenic growth factors during embryonic development and disease pathogenesis.^[^ ^19^ ^]^ However, the relationship between DDR1 and HIF‐1α is largely unclear. HIF‐1α mRNA is constantly translated into protein in cells, while due to persistent degradation by the ubiquitin‒proteasome system, the protein level of HIF‐1α remains extremely low under normal oxygen tension.^[^ ^26^ ^]^ Our results demonstrated that DDR1 upregulation could delay the degradation of HIF‐1α and thus raise its protein level. These data suggest that posttranscriptional modifications may contribute to DDR1‐induced HIF‐1α protein expression.

In this study, we demonstrated that DDR1 inhibited the ubiquitination and degradation of HIF‐1α in GC cells, probably via direct binding between DDR1 and HIF‐1α, proposing a novel mechanism by which DDR1 regulates the dynamic equilibrium of HIF‐1α protein expression. Furthermore, our data showed that the PAS domain of HIF‐1α directly interacted with DDR1. Indeed, the PAS domain is involved in a variety of regulatory and sensory functions, such as the stability, protein interactions, and nuclear localization of HIF‐1α.^[^ ^44^ ^]^ Further experiments demonstrated that the PAS domain was vital for DDR1‐induced angiogenesis. Collectively, these results provide insights into a novel mechanism by which DDR1 affects the cellular HIF‐1α level.

In addition to angiogenesis, some studies have demonstrated that DDR1 enhances metastatic capacity, but the mechanism is not well understood.^[^ ^45^ ^]^ Actin cytoskeleton reorganization is closely related to tumor cell migration, and DDR1 is suggested to be correlated with actin cytoskeleton remodeling, although it plays distinct roles in different cell types.^[^ ^28^, ^29^, ^46^ ^]^ Some studies have demonstrated that DDR1 stimulates the formation of linear invadosomes and actin stress fibers in breast cancer cells,^[^ ^28^, ^30^ ^]^ while Yeh et al. reported that DDR1 inhibits Rac1/Cdc42 activity to decrease the F‐actin content in Madin‐Darby canine kidney (MDCK) cells.^[^ ^47^ ^]^ Our study first demonstrated that DDR1 mediated cytoskeletal reorganization by increasing the accumulation of actin stress fibers and further enhancing the metastatic capacity of GC cells. Actin cytoskeleton reorganization is commonly coupled with EMT, which is an initial step for tumor metastasis cascades.^[^ ^48^ ^]^ We also found that DDR1 induced EMT in GC cells. However, the mechanism by which DDR1 regulates actin cytoskeleton reorganization and EMT is still unclear. RhoA and its downstream kinase, ROCK1, are known to be important regulators extensively involved in actin cytoskeleton reorganization.^[^ ^49^ ^]^ Gilkes et al. suggested that HIF‐1α was an activator of RhoA/ROCK1 signaling.^[^ ^50^ ^]^ Our study further provides evidence that DDR1 promotes actin cytoskeleton reorganization through HIF‐1α/RhoA/ROCK1 signaling in GC cells and clarifies a novel mechanism for tumor metastasis in GC.

The past decade has seen little progress in anticancer drugs for GC, leading to limited survival improvements lagging those in other malignancies.^[^ ^51^ ^]^ At present, trastuzumab is the only effective targeted therapeutic drug for GC in clinical practice and can improve the prognosis of HER2‐positive GC patients.^[^ ^52^ ^]^ However, only 10%–20% of GC cases show HER2 overexpression or amplification.^[^ ^53^ ^]^ Thus, there is an urgent need to explore novel therapeutic targets for GC. PDX and organoid models reproduce the genetic and phenotypic heterogeneity of the original tumors and mimic the biological characteristics much better than in vitro culture models.^[^ ^54^ ^]^ Therefore, we established PDX and organoid models of GC, as we have previously described, and tested the effect of pharmacological inhibition of DDR1 with 7rh benzamide.^[^ ^55^ ^]^ Treatment with 7rh benzamide efficiently inhibited tumor angiogenesis and growth in the PDX models. In addition, our organoid model results showed that 7rh benzamide attenuated the reorganization of the actin cytoskeleton, consistent with the in vitro results. Further clinical trials should be performed to validate the therapeutic effect of 7rh benzamide in GC. Collectively, these results provide solid evidence for DDR1 as a treatment target for GC.

Regarding the limitations, this work does not include a pre‐clinical trial using a DDR1 inhibitor or DDR1 monoclonal antibody to block the interaction between DDR1 and HIF‐1α. Incorporating such a trial would provide highly meaningful and convincing evidence of the therapeutic effect in GC patients through the proposed molecular mechanism. Additionally, many E3 ligases are reported to interact with HIF‐1α and participate in its degradation, including VHL, RACK1, FBW7, MDM2, SIAH1/3, and CHIP, among others.^[^ ^56^ ^]^ The binding of DDR1 with HIF‐1α could potentially act as an antagonist, preventing E3 ligase‐mediated HIF‐1α degradation and stabilizing HIF‐1α. Further studies should investigate which E3 ligase can be antagonized and define the extent of this antagonization. This will lead to a deeper understanding of DDR1's role in regulating GC malignancy.

In summary, our results demonstrated that DDR1 triggered GC progression by directly binding to HIF‐1α, which subsequently suppressed its ubiquitin‐mediated degradation. Through HIF‐1α/RhoA/ROCK1 signaling, DDR1 promoted cytoskeleton reorganization to induce tumor metastasis. Pharmacological inhibition of DDR1 retarded tumor progression in PDX and organoid models. This work revealed the intracellular mechanism of DDR1 in driving GC progression and identified the DDR1 antagonist 7rh benzamide as a promising therapeutic option for GC patients.

## 4 Experimental Section

### Patients and Tissue Specimens

Paraffin‐embedded GC specimens were obtained from 182 GC patients diagnosed with histologically proven GC between 2012 and 2014 in this study. The GC patient information and follow‐up data were obtained from the gastric cancer database at the First Affiliated Hospital of Sun Yat‐sen University (FAHS). In addition, fresh tumor tissues and adjacent normal tissues were collected from 40 GC patients who received radical gastrectomy in the FAHS.

All specimens were collected with written informed consent. This study was approved by the Ethical Review Committee of the FAHS (approval numbers: [2021]−497) and the Seventh Affiliated Hospital of Sun Yat‐sen university (approval numbers: KY‐2020‐042‐01). The 8th edition of the tumor‐node‐metastasis (TNM) Classification of Malignant Tumors was used for the staging of GC cases.

### Cell Culture

HEK293T cells were grown in Dulbecco's modified Eagle's medium (DMEM) containing 10% fetal bovine serum (FBS) (Gibco) and 1% penicillin‒streptomycin (Invitrogen). HGC27, AGS and MKN74 cells were cultured in RPMI‐1640 medium supplemented with 10% FBS (Gibco) and 1% penicillin‐streptomycin (Invitrogen). HUVECs were isolated and cultured in gelatine‐coated cell culture dishes in M199 medium (Invitrogen) containing 20% FBS, 10 µg L^−1^ human basic fibroblast growth factor (bFGF) (R&D Systems) and 1% penicillin‒streptomycin (Invitrogen). The cell lines used in this study were validated by short tandem repeat profiling. All the cells were regularly screened for mycoplasma contamination and used only when negative.

### Transfection and Vectors

Plasmids expressing HA‐tagged DDR1 (pEZ‐M07‐HA‐DDR1), Flag‐tagged HIF‐1α (pEZ‐M14‐Flag‐HIF‐1α) and the corresponding deletion mutants were purchased from GeneCopoeia (Guangzhou, China). Flag‐tagged HIF‐1α deletion mutants were generated by site‐directed mutagenesis using a QuikChange II XL Site‐Directed Mutagenesis Kit (Agilent Technologies). H1, H2 and H3 deletion mutants were generated by individually deleting the PAS (85‐298), NODD (401‐531), and ID (575‐786) motifs, respectively, from the HIF‐1α sequence. Plasmids were transfected into cells at 70%−80% confluence cultured in 6‐well plates with Lipofectamine 3000 reagent (Invitrogen, Carlsbad, California, USA). Full‐length DDR1 cDNA was inserted into the lentiviral vector pEZ‐Lv201 (GeneCopoeia). Lentiviral particles expressing pEZ‐Lv201‐DDR1 and empty pEZ‐Lv201 were used for transduction of HGC27 and AGS cells according to the manufacturer's instructions. HIF‐1α silencing was performed by inserting the sequences 5′‐UGCUCUUUGUGGUUGGAUCUA‐3′ (HIF‐1α‐shRNA‐1) and 5′‐CCGCUGGAGACACAAUCAUAU‐3′ (HIF‐1α‐shRNA‐2) into the psi‐LVRU6H lentiviral vector (GeneCopoeia, China) vector. Cells were incubated with puromycin (2 µg ml^−1^) or hygromycin (200 µg ml^−1^) for selection of stable cells expressing DDR1 or HIF‐1α‐shRNA.

### CRISPR/Cas9‐Mediated Knockout of the DDR1 Gene

CRISPR/Cas9 gene editing was adopted to knock out DDR1 expression in human HGC27 and AGS cells. The single‐guide RNA (sgRNA) targeting DDR1 (sgRNA sequence: ATCAGGAGCTATGGGACCAG) was inserted into the lentiviral vector pCRISPR‐LvSG06 (GeneCopoeia, China). Then, lentiviral particles were used for transduction in the presence of 10 µg ml^−1^ polybrene. Stable cell lines were selected by incubation with puromycin (3 µg ml^−1^) for 2 weeks.

### Immunohistochemistry

IHC analysis was conducted on paraffin‐embedded human GC tissues and subcutaneous tumor tissues from mice as previously described.^[^ ^57^ ^]^ The primary antibodies used for IHC were listed in Table S4 (Supporting Information). Visualization was carried out with DAB (DAKO, #K5007) and counterstained with hematoxylin. Images were captured with an Olympus BX63 microscope (Olympus, Japan). The IHC scores were determined by the proportion and intensity of positive staining in tumor cells. Samples with scores ≥ 8 were deemed to have high expression, and those with a score of 0–8 were considered to have low expression. Two pathologists blinded to the clinicopathological characteristics independently selected 5 high‐power fields in the stained slides and assessed the proportions and intensities.

### Western Blotting

Standard western blot analysis was performed as previously described.^[^ ^57^ ^]^ The primary antibodies for immunoblotting in Table S5 (Supporting Information) was listed. Finally, the membranes were visualized using enhanced chemiluminescence reagent (Millipore, #WBKLS0100) with an imaging system (Tanon 5200 Multi, China).

### Tumor Cell Migration and Invasion Assays

Cell migration and invasion assays were conducted in 24‐well plates using modified Boyden chambers (8‐mm pore size; BD Biosciences). Cells (5×10^4^) were suspended in serum‐free medium and seeded into the upper chambers with (invasion assay) or without (migration assay) a Matrigel coating on the membrane. The lower chambers were filled with medium containing 10% FBS. After incubation for 24 h, cells remaining in the upper chambers were removed. The migrated cells were fixed with 4% formaldehyde and then stained with 0.5% crystal violet. The migrated and invaded cells were counted in five random fields at 100× magnification.

### Wound Healing Assay

AGS and HGC27 cells were cultured in 6‐well plates (5 × 10^5^ cells well^−1^) and grown to 80% confluence. A scratch was made in the cell layer with a sterile pipette tip, and the detached cells were removed by replacement of the medium with RPMI‐1640 medium containing 1% FBS. Cell movement into the wound area was evaluated with a phase‐contrast microscope (Nikon, Japan) 48 h after the scratch was made.

### Co‐IP Assay

Cells were cultured in 10 cm dishes and lysed with IP lysis buffer (Thermo Fisher). The supernatants obtained from cell lysates after centrifugation were incubated with primary antibodies overnight. Then, the protein samples were incubated with Pierce Protein A/G Magnetic Beads (Thermo Fisher) at 4 °C for 2 h and washed with IP lysis buffer three times. Proteins were collected using elution buffer (Thermo Fisher). Finally, the protein samples were analyzed by SDS‒PAGE and immunoblotted with the corresponding antibodies. The primary antibodies used for the co‐IP assay are listed in Table S6 (Supporting Information).

### RhoA Activity Assay

After washing with ice‐cold PBS 2 times, cells were lysed with cell lysis buffer. Then, the cell lysates were centrifuged at 14 000 rpm for 5 min at 4 °C. The proteins in the supernatants were collected and incubated with agarose‐bound rhotekin‐RBD beads at 4 °C for 45 min by using the Rho Activation Assay Biochem Kit (#BK036, Cytoskeleton). The beads were pelleted by centrifugation and washed three times in ice‐cold Mg^2+^ lysis buffer (MLB). The level of active RhoA was measured by immunoblotting using an anti‐RhoA antibody.

### Tube Formation and HUVEC Migration Assays

For tube formation assays, Matrigel matrix (Corning, #356 234) was utilized to coat the wells of 24‐well plates and incubated at 37 °C for 30 min. HUVECs (1×10^5^) were seeded in Matrigel‐coated 24‐well plates and treated for 6 h at 37 °C with conditioned medium from GC cells transfected with different vectors. Images of the capillary tube structure were captured by phase contrast microscopy. The tubes were counted to evaluate the tube formation ability. For HUVEC migration assays, cells (3×10^4^) were seeded into Transwell chambers (8‐mm pore size; BD Biosciences) in a 24‐well plate. After treatment with conditioned medium for 24 h, the migrated HUVECs were stained with 0.5% crystal violet and counted. HUVECs at passages 2–6 were utilized in the study.

### Enzyme‐Linked Immunosorbent Assay

A human VEGF‐A enzyme‐linked immunosorbent assay (ELISA) kit (Elabscience, #E‐EL‐H0111) was used to measure the secreted VEGF‐A protein level according to the manufacturer's instructions.

### Cell Immunofluorescence

Cells seeded on coverslips were fixed with 4% paraformaldehyde for 15 min at room temperature and permeabilized in 0.2% Triton X‐100 for 5 min. Then, after blocking with blocking buffer (PBS with 5% BSA) for 30 min at RT, the cells were incubated with antibodies overnight at 4 °C followed by secondary antibodies for 1 h at RT. The primary antibodies for cell IF were listed in Table S7 (Supporting Information). Thereafter, samples were counterstained with DAPI for 5 min to stain the nuclei. Images were captured with an LSM780 confocal microscope (Zeiss).

### RNA Extraction, Quantitative Real‐Time PCR and Sequencing Analysis

Total RNA was extracted with RNAiso Plus (Takara, Japan) as it was previously described.^[^ ^57^ ^]^ cDNA synthesis was conducted with a reverse transcription kit (TAKARA, Japan). RT‐PCR analysis was conducted on an HT 7900 instrument (Applied Biosystems) in 10 µl reaction mixtures with SYBRVR Green qPCR SuperMix (Invitrogen). The primer sequences used are listed in Table S8 (Supporting Information). Relative mRNA expression levels were calculated as fold changes using the 2^−ΔΔCt^ method.

Another 131 GC samples from FAHS were utilized for sequencing analysis. The sequences of the primers used to amplify regions containing previously reported DDR1 mutations were as follows: forwards primer GAGCTGACGGTTCACCTCTC, reverse primer AATGTCAGCCTCGGCATAAT. Then, the polymerase chain reaction products were purified and sequenced.

### Animal Studies

The protocols of animal studies were in accordance with the guidelines of the First Affiliated Hospital of Sun Yat‐sen University. The animal studies were approved by the Ethical Review Committee of the FAHS (approval numbers: [2021]−165). Nude mice were obtained from GemPharmatech Co., Ltd. (Guangdong, China). For the tumor xenograft model, AGS cells (5×10^6^) transduced with the indicated vectors were utilized to establish subcutaneous tumors in the right axillae of nude mice (female, 5–6 weeks old) (n = 6 per group). Tumor growth was monitored with callipers every three days, and tumor volume was quantified as follows: V (mm^3^) = [Length×Width^2^] ×0.5. After 4 weeks, mice were euthanized, and tumors were collected, weighed, and preserved for further analysis.

Female BALB/c nude mice were used to establish lung metastasis models. AGS cells (1×10^6^) transduced with the indicated vectors were injected into the tail vein. After 8 weeks, the lungs of mice from different groups (n = 6 per group) were collected and fixed in 4% paraformaldehyde. Then, serial lung sections stained with hematoxylin and eosin (H&E) were used to measure the metastatic nodules. Experiments were blinded to the people conducting marker procedure.

### Establishment of PDX Models

To establish PDX models, fresh tumor tissues were obtained immediately after removal from GC patients and cut into 3×3×3 mm^3^ pieces, which were further subcutaneously implanted into the flanks of nude mice. The successfully established PDXs were regarded as passage 1 (P1). Then, the mice were sacrificed, and the xenografts were collected and transplanted into other nude mice to obtain the next passage of tumors. Nude mice with P3 tumors were used to evaluate the efficacy of 7rh benzamide (Sigma–Aldrich, #SML1832). When the tumor volume reached ≈200 mm^3^, mice were randomly assigned into two groups receiving 7‐rh benzamide (25 mg kg^−1^, every day, oral gavage) or vehicle. Tumor growth was measured with callipers every three days. All mice were sacrificed four weeks later, and the xenografts were harvested, weighed and processed for immunohistochemical and immunoblot analyses.

### Organoid Culture

The establishment and culture of GC organoids were performed as we previously described.^[^ ^55^ ^]^ Human GC tissues for organoid establishment were collected immediately after removal during surgery. All patients signed informed consent forms. GC specimens were preserved in Advanced DMEM/F12 supplemented with 1% penicillin‒streptomycin (Invitrogen) and cut into pieces of 3 mm^3^. The tumor pieces were digested into solutions by incubation with Advanced DMEM/F12 medium and 1 mg ml^−1^ type IV collagenase (Sigma–Aldrich) at 37 °C for 1 h. After digestion, samples were strained through a 70‐µm filter (Falcon; #352 350) and resuspended in medium mixed with Matrigel at a ratio of 2:1. Organoids were passed every 2–3 days. At the approximate size and confluence, organoids were collected and fixed with 4% methanol‐free paraformaldehyde (PFA) for IF analysis.

### RNA‐Seq Analysis and Bioinformatics

The TRIzol reagent was used to lyse cell, then the total RNA was extracted for high‐throughput sequencing. The Beijing Genomics Institute was responsible for library construction and high‐throughput RNA sequencing. The limma package was utilized to conduct differential gene expression analysis between experimental conditions. Each condition was represented by three independent biological replicates. DEGs (differentially expressed genes) were determined by > 1‐fold change in gene expression with adjusted *P*< 0.05 and were visualized using volcano plot. The upregulated differentially expressed genes within DDR1 overexpressed cells were selected for GO enrichment analysis. Gene set enrichment analysis (GSEA) was also performed for RNA‐seq data, against signatures in the Molecular Signatures Database.

### Gene Expression Analysis in Public Databases

We extracted the RNA‐Seq expression data (HTSeq‐FPKM), clinicopathological features, and prognostic data of GC patients from the TCGA‐STAD database. To compare collagen receptor expression, a log2 (TPM+1) transformation was applied to standardize the mRNA expression levels. The GSE51575 and GSE13861 datasets downloaded from the PubMed GEO database were also obtained to evaluate DDR1 expression in gastric cancer and adjacent normal tissues. For correlation analysis between DDR1 and VEGF gene expression in the cohort of GC patients, the data for the gene expression matrix were extracted from TCGA‐STAD datasets, and correlations were analyzed in R using two‐tailed Spearman correlation analysis. To investigate the correlations between the expression of DDR1 and VEGFs in GC, it was extracted data from the TCGA‐STAD dataset and used two‐tailed Spearman correlation analysis. The visualization of the correlation matrix was conducted with the R packages “ggplot2” and “ggcorrplot”.

### Statistical Analysis

The chi‐square test was used to compare differences between 2 groups of categorical variables. For continuous variables, parametric or nonparametric tests were adopted. One‐way analysis of variance was utilized for comparisons among three or more groups. The statistical analyses used are described in figure legend. Data are presented as the mean ± SEM of at least three independent experiments. GraphPad Prism 8 (GraphPad Software Inc., USA), SPSS 22.0 (SPSS Inc., USA) or R software were used for statistical analyses. Statistical significance was deemed to be indicated by *P*< 0.05.

## Conflict of Interest

The authors declare no conflict of interest.

## Author Contributions

Z.W.W., J.L., L.Z., and D.J.Y. contributed equally to this work. L.Z., Y.H., W.S., and B.W. participated in the study design. Z.W., L.Z., J.L., and B.W. performed the in vitro experiments. Z.W., W.C., and D.Y. conducted the data analyses. Z.W., L.Z., and W.L. performed the PDX and nude mice experiments. W.C., L.Z., and H.Z. performed the organoid experiments. L.Z., Z.W., L.Z., and D.Y. wrote the manuscript, Z.W. revised the manuscript. W.S. and Z.W. interpreted the data and revised the manuscript. All authors read and approved the final manuscript. Funding for the research was provided by L.Z., Y.H., and W.S.. The order of the co–first authors was assigned based on the relative contributions of these individuals.

## Ethics

All patients in this study signed an informed consent form. This study was approved by the Ethical Review Committee of the First Affiliated Hospital of Sun Yat‐sen University involving Human Subjects and the use of mice.

## Supporting information

Supporting Information

## Data Availability

The data that support the findings of this study are available from the corresponding author upon reasonable request.
