# Supplementary material for: DDR1 Drives Malignant Progression of Gastric Cancer by Suppressing HIF‐1α Ubiquitination and Degradation
Source: Adv Sci (Weinh). 2024 Jul 18;11(35):2308395. doi: 10.1002/advs.202308395 (PMC11425230; doi:10.1002/advs.202308395)
Supplement: Supplementary file 1 — Supporting Information [file ADVS-11-2308395-s001.docx]

**
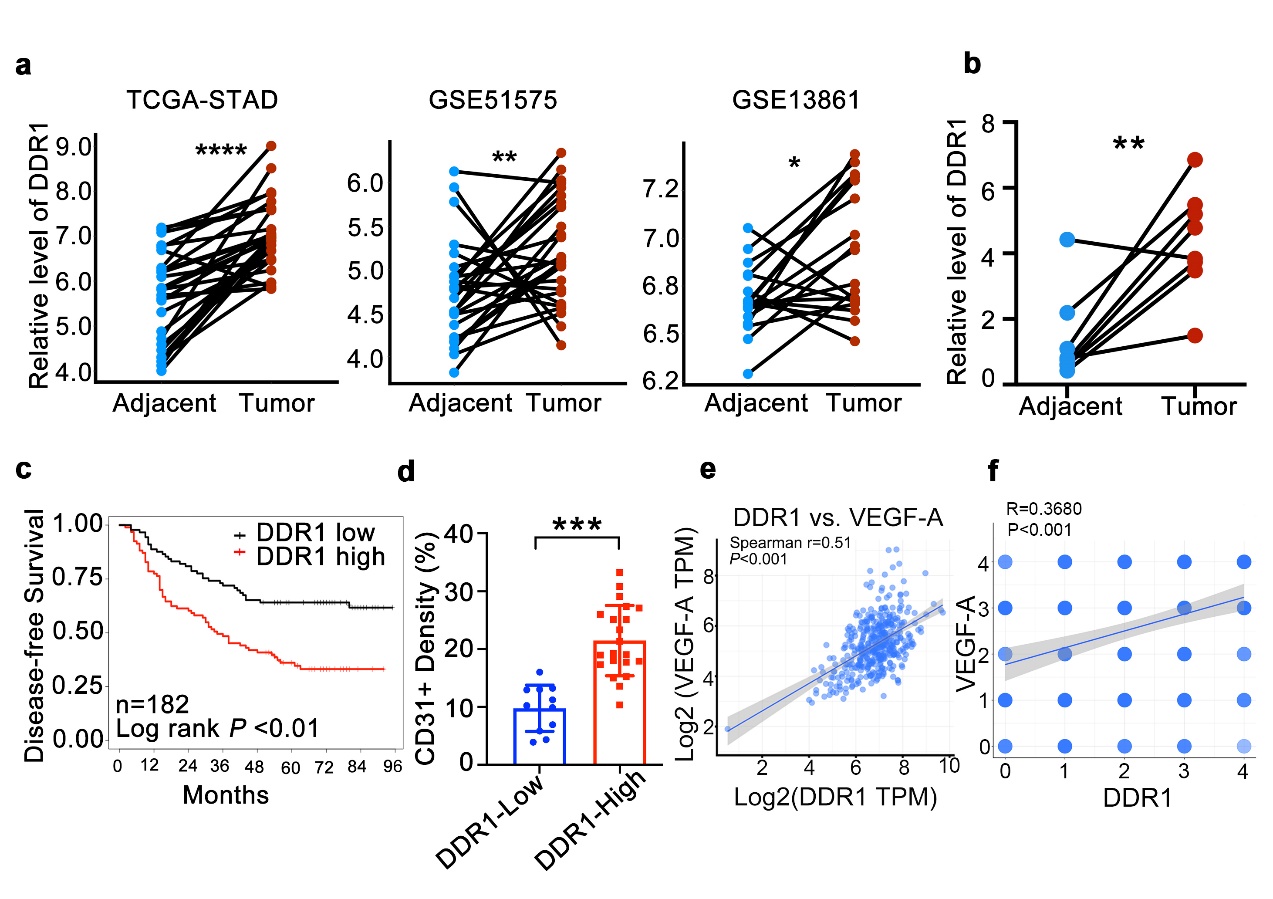
Supplementary Figures**

**Figure S1. DDR1 expression is increased in tumour tissues and correlated with worse survival in GC.**

**(a)** The mRNA levels of DDR1 in tumours were higher than those in the adjacent normal tissues based on data from TCGA-STAD cohort, GSE51575 and GSE13861 datasets. **(b)** RT-PCR analysis was used to determine the mRNA expression of DDR1 in 8 paired tumor and adjacent non-tumor tissues from GC patients at the First Affiliated Hospital of Sun Yat-sen University (FAHS). **(c)** Kaplan-Meier curves showed that GC patients with high DDR1 expression exhibited worse disease-free survival (DFS) based on a cohort from FAHS (log rank test; n=182). **(d)** CD31 density was quantified in DDR1-High and DDR1-Low GC tissues (n=32) by IF staining. **(e)** Spearman correlation analysis revealed that DDR1 was positively correlated with VEGF-A expression based on data from TCGA-STAD cohort. All data were interpreted as means ± SEM from three independent experiments. The *P* values in panels (a), (b) were calculated using paired *t*-test. The *P* values in panels (d) were calculated using Student’s *t*-test. **P* < 0.05, ****P* < 0.001.

**
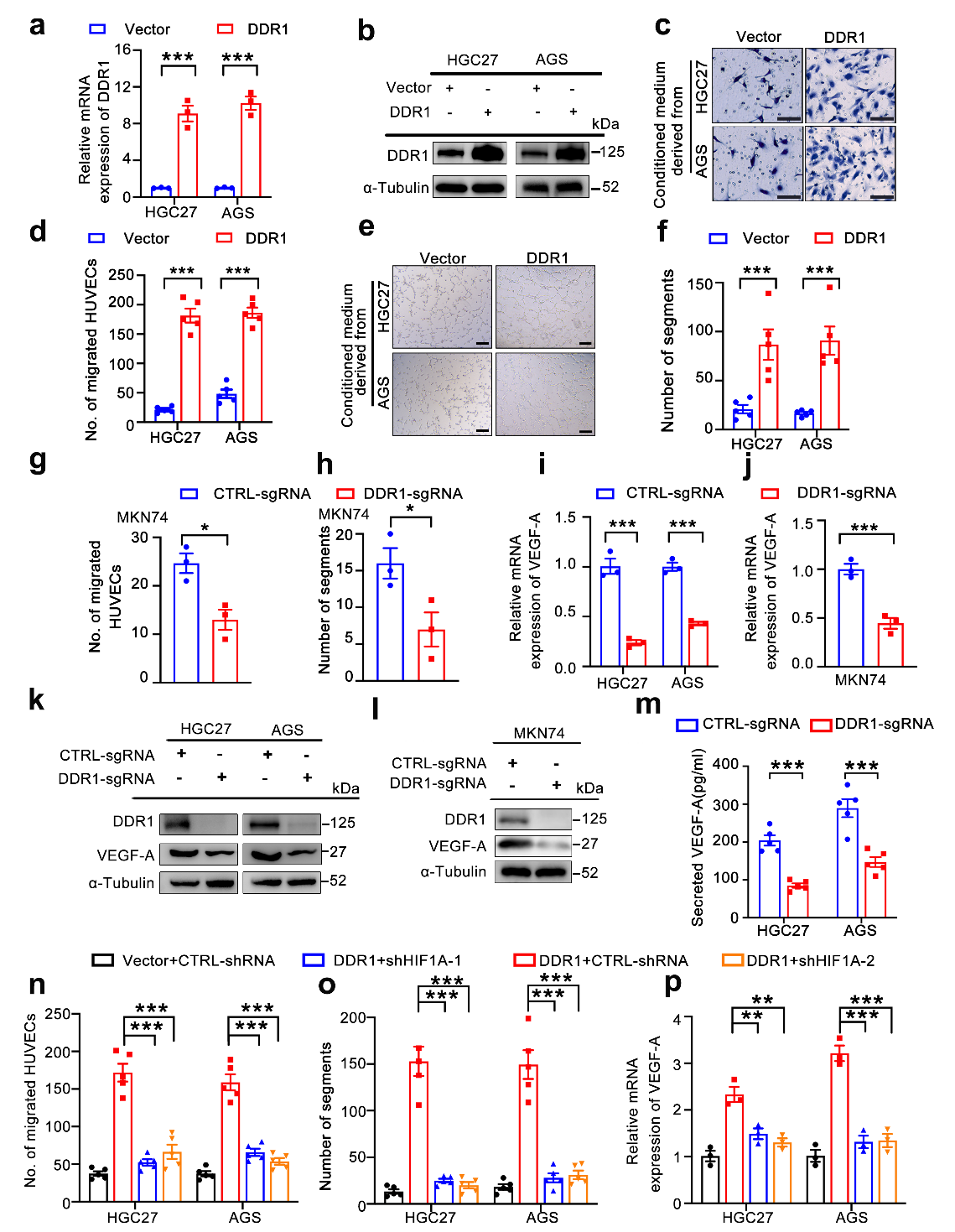
**

**Figure S2. HIF1A knockdown represses DDR1-induced GC angiogenesis in vitro.**

(**a, b**) The mRNA (a) and protein (b) levels of DDR1 were measured by RT-PCR and western-blot analysis in HGC27 and AGS cells transduced with DDR1-expressing or control vector. (**c, d**) DDR1 overexpression in GC cells promoted the migration of HUVECs. Representative images (c) and quantification (d) of migrated HUVECs incubated with conditioned medium (CM) from HGC27 or AGS cells transduced with DDR1-expressing or control vector. Scale bar: 100μm. (**e, f**) DDR1 overexpression in GC cells augmented the capillary tube formation of HUVECs. Representative images (e) and quantification (f) of the capillary tube formation assays of HUVECs incubated with CM from HGC27 or AGS cells transduced with DDR1-expressing or control vector. Scale bar: 200μm. (**g, h**) Knockout of DDR1 in MKN-74 cells suppressed the migration and tube formation of HUVECs. (**i, j**) RT-PCR analysis suggested that DDR1 knockout inhibited VEGF-A expression in HGC-27, AGS and MKN-74 cells. (**k, l**) DDR1 knockout inhibited VEGF-A expression in HGC-27, AGS and MKN-74 cells. Western blot analyses were performed to evaluate the VEGF-A expression in GC cells transfected with Control sgRNA and DDR1-sgRNA. (**M**) DDR1 knockout attenuated VEGF-A secretion in AGC and HGC-27 cells. Secreted VEGF-A levels in the CM of AGC and HGC-27 cells transfected with Control sgRNA and DDR1-sgRNA were measured by ELISA. (**n, o**) Silencing of HIF1A in DDR1-overexpressed GC cells inhibited the migration and tube formation of HUVECs. (**p**) Knockdown of HIF1A suppressed VEGF-A expression in DDR1-overexpressed GC cells. RT-PCR was performed to measure the mRNA levels in HGC27 or AGS cells transfected with the indicated vectors. All data are presented as the mean ± SEM from three independent experiments. The *P* values in panels (a), (d), (f-j) and (m) were calculated using Student’s *t*-test. The *P* values in panels (n-p) were calculated using one-way ANOVA. **P* < 0.05, ***P* < 0.01, ****P* < 0.001.


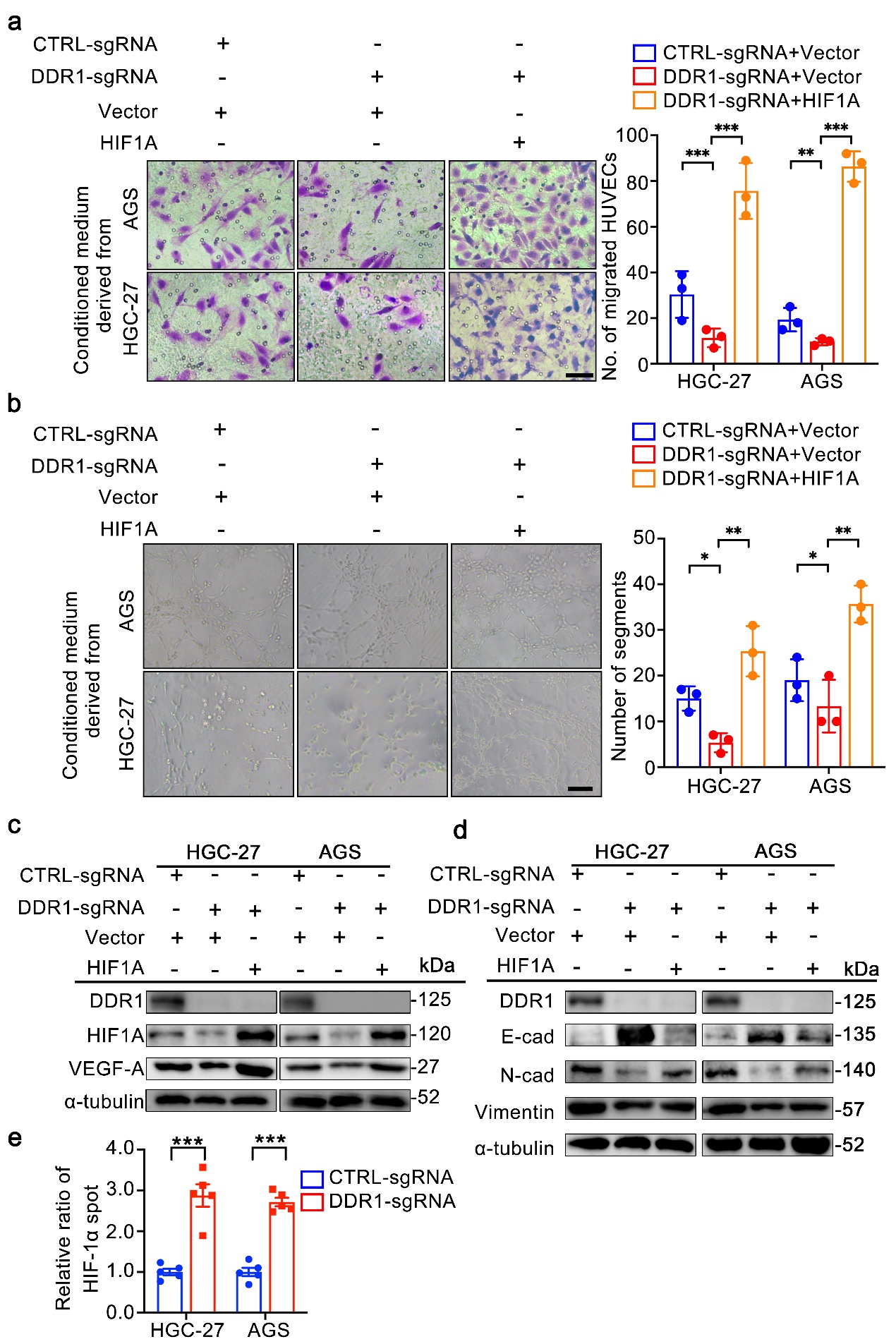


**Figure S3. HIF1A overexpression restored the reduction in angiogenesis and metastasis caused by DDR1 knockout in vitro.**

(a) *HIF1A* overexpression promoted the migrations of HUVECs in DDR1-knockout GC cells. Representative images (left) and quantifications (right) of migrated HUVECs incubated with CM from HGC27 or AGS cells transduced with the indicated vectors. Scale bar: 100μm. (b) HIF1A overexpression promoted capillary tube formation of HUVECs in DDR1-knockout GC cells. Representative images (left) and quantification (right) of the capillary tube formation assays of HUVECs incubated with CM from HGC27 or AGS cells transduced with the indicated vectors. Scale bar: 200μm. (c) *HIF1A* overexpression restored VEGF-A expression in DDR1-knockout GC cells. Western blot analysis was performed to measure the protein level of VEGF-A in HGC27 and AGS cells transfected with the indicated vectors. (d) *HIF1A* overexpression restored EMT markers (E-cadherin, N-cadherin and Vimentin) in DDR1-knockout GC cells. Western blot analysis was performed to measure the protein level of E-cadherin, N-cadherin and Vimentin in HGC27 and AGS cells transfected with indicated vectors. (e) *DDR1* knockout increased the colocalization of ubiquitin and HIF-1α in GC cells. Quantification of the colocalization of ubiquitin (green) and HIF-1α (red), as determined by IF analysis. All data are presented as the mean ± SEM from at least three independent experiments. The *P* values in panels (e) were calculated using Student’s *t*-test. The *P* values in panels (a-b) were calculated using one-way ANOVA. **P* < 0.05, ***P* < 0.01, ****P* < 0.001.

**
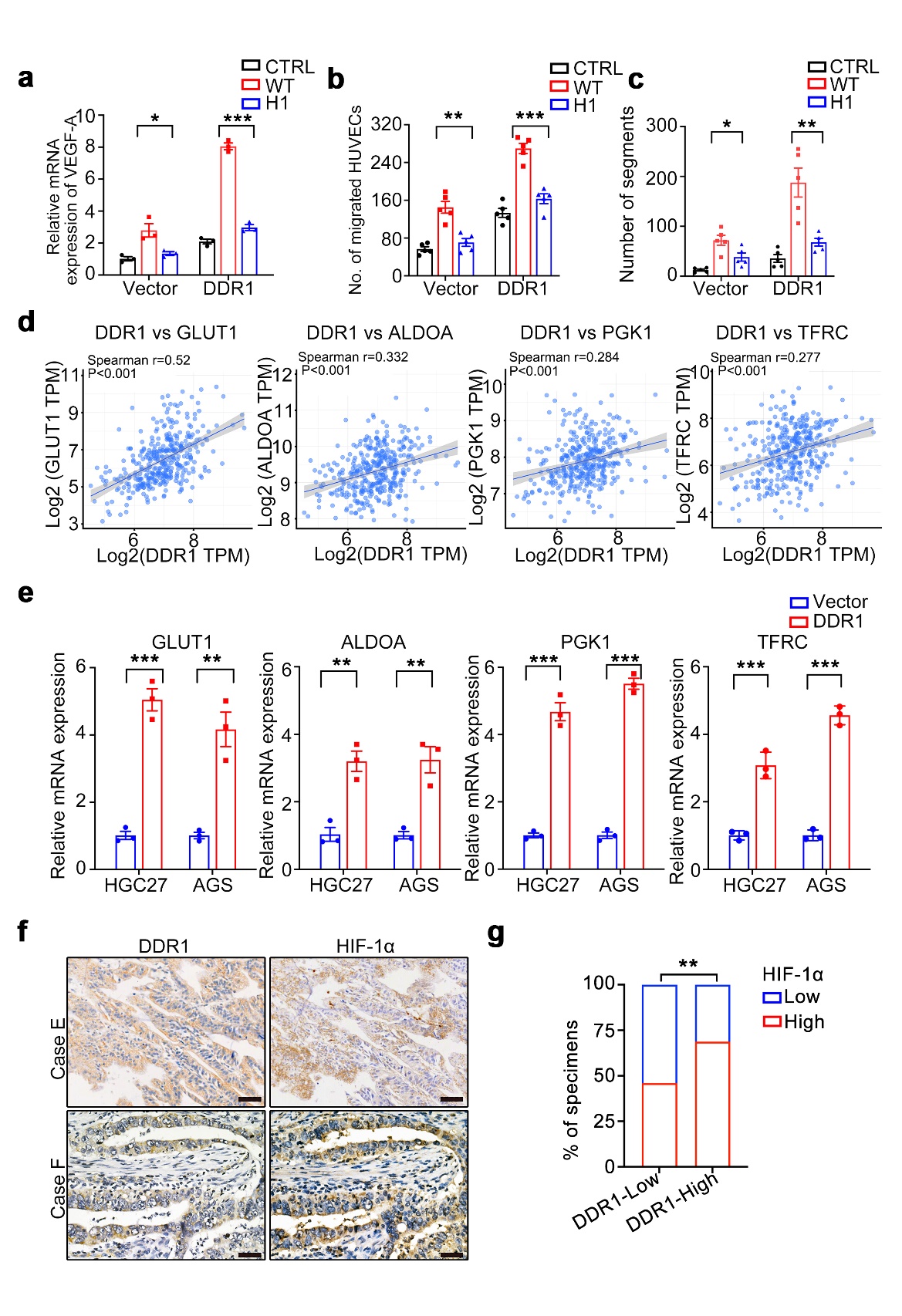
**

**Figure S4. DDR1 is positively correlated with HIF-1α level and its target genes in GC.**

**(a)** The upregulation of VEGF-A induced by DDR1 was attributed to the PAS domain of HIF-1α. The mRNA levels of VEGF-A in cells transfected with control vector and vectors expressing wild-type (WT) or PAS deletion mutants (H1) of HIF-1α-Flag were measured by RT-PCR. **(b)** The PAS domain of HIF-1α was required for DDR1-induced migration of HUVECs. Quantification of the migrated HUVECs cultured with CM from cells transfected with control vector, vectors expressing WT or H1 deletion mutants of HIF-1α-Flag. **(c)** The PAS domain of HIF-1α was responsible for DDR1-induced capillary tube-formation of HUVECs. Quantifications of the tube-formation of HUVECs cultured with CM from cells transfected with control vector, vectors expressing WT or H1 deletion mutants of HIF-1α-Flag. **(d)** Spearman correlation analysis observed positive correlations between DDR1 and target genes of HIF-1α based on the data from TCGA-STAD datasets. **(e)** DDR1 was positively correlated with the target genes of HIF-1α. RT-PCR was used to measure the mRNA levels of HIF-1α target genes, including *GLUT1, ALDOA, PGK1* and *TFRC* in HGC27 and AGS cells transduced with DDR1-expressing or control vector. **(f, g)** DDR1 was positively correlated with HIF-1α expression in GC tissues. Representative images (f) and quantification (g) of DDR1 and HIF-1α determined by IHC based on the cohort from FASH (n=182). Scale bars: 25um. All data were interpreted as means ± SEM from three independent experiments. The *P* values in panels (a), (b), (c) were calculated using one-way ANOVA. The *P* values in panels (e) were calculated using Student’s *t*-test. The *P* values in panels (g) were calculated using χ^2^ test. **P* < 0.05, ***P* < 0.01, ****P* < 0.001.

**
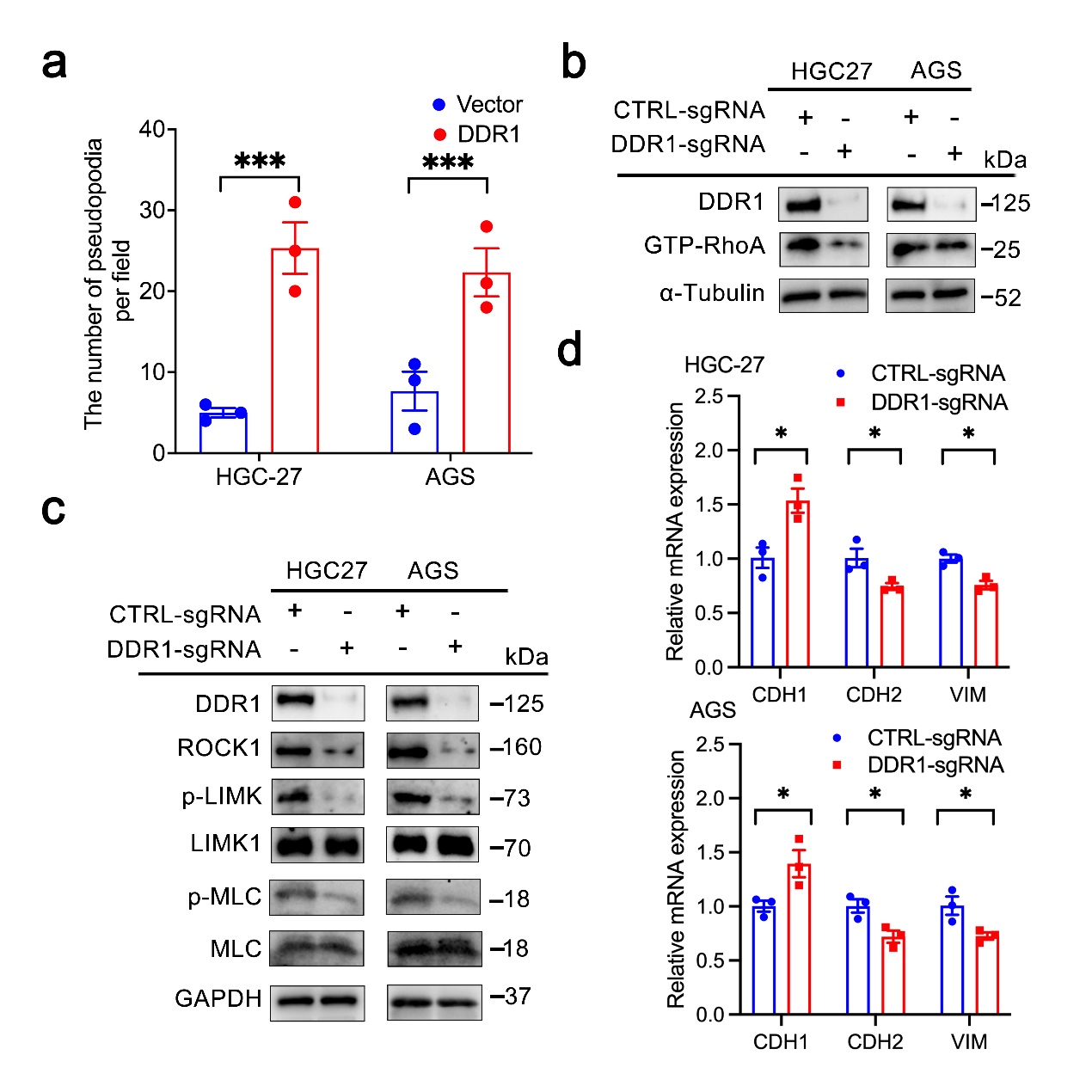
**

**Figure S5. DDR1 regulates the RhoA/ROCK1 signaling in GC cells.**

**(a)** Overexpression of DDR1 induced reorganization of the actin cytoskeleton in GC cells, as evidenced by an increase in the number of pseudopodia. Quantification of pseudopodia in HGC27 and AGS cells transfected with DDR1-expressing or control vector. **(b)** *DDR1* knockout markedly decreased RhoA activity in GC cells. RhoA activity in HGC27 and AGS cells transfected with Control sgRNA and DDR1-sgRNA was determined by a GTP-RhoA pulldown assay using GST-Rhotekin-RBD. **(c)** DDR1 regulated ROCK1 expression and the phosphorylation of LIMK and MLC in GC cells. Western blot analysis was used to measure ROCK1, p-LIMK, LIMK1, p-MLC and MLC levels in HGC27 and AGS cells transfected with the indicated vectors. **(d)** DDR1 regulated the EMT process in GC cells. The mRNA levels of E-cadherin, N-cadherin and Vimentin were analyzed by RT-PCR in HGC27 and AGS cells transfected with the DDR1-expressing or control vector. All data were interpreted as means ± SEM from three independent experiments. The *P* values in panels (a), (d) were calculated using Student’s *t*-test. **P* < 0.05, ****P* < 0.001.

**
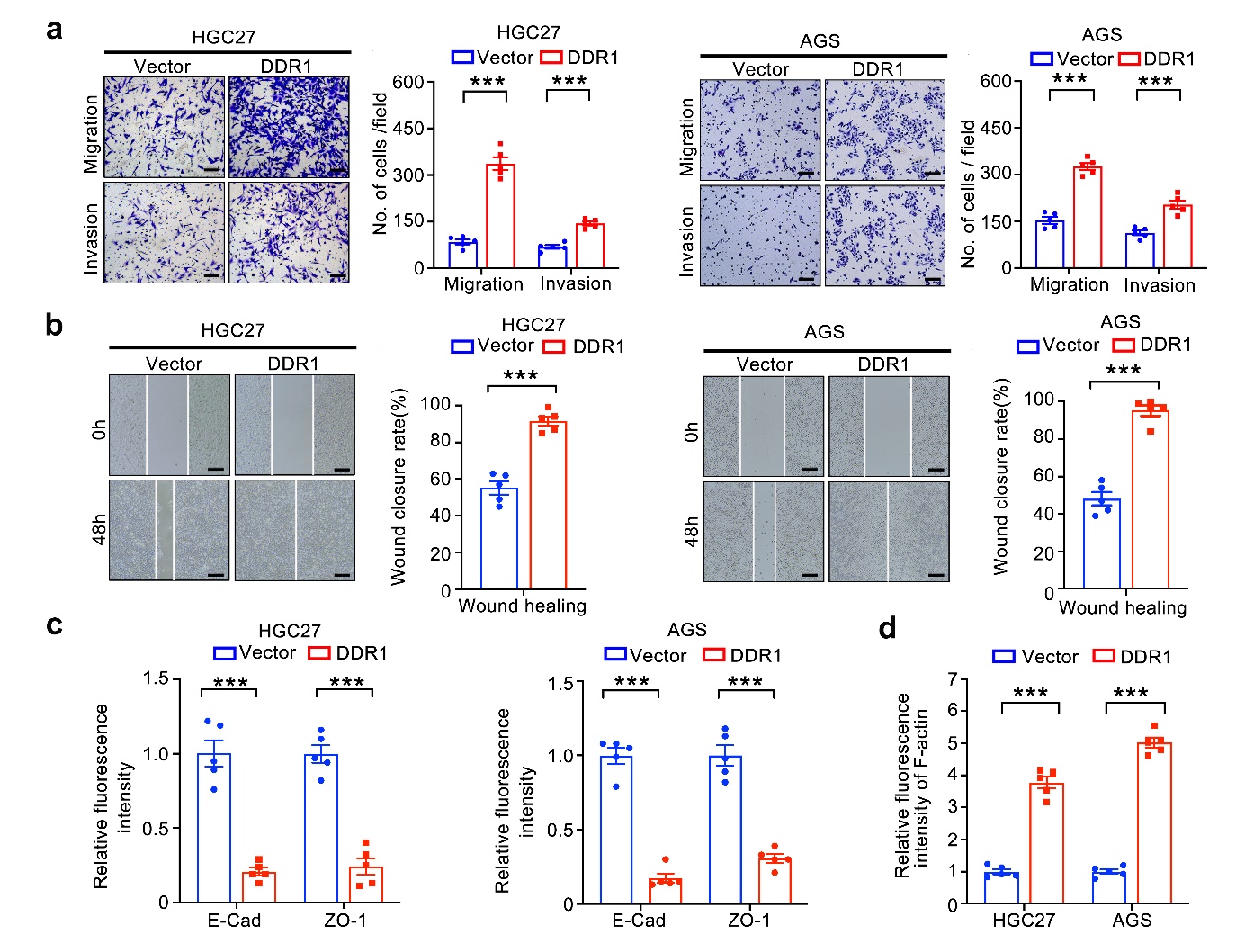
Figure S6. DDR1 promotes the migration and invasion of GC cells *in vitro*.**

**(a)** DDR1 increased the migratory and invasive capabilities of GC cells. Transwell migration and Matrigel invasion assays were performed on HGC27 and AGS cells transduced with DDR1-expressing or control vector, and representative images and statistical analysis were obtained. Scale bar: 100 μm. **(b)** The wound healing assays suggested that DDR1 overexpression in GC cells enhanced their motilities. Scale bar: 200μm. **(c)** DDR1 downregulated E-cadherin and ZO-1 expression in GC cells. The quantifications of fluorescence intensity of E-Cadherin and ZO-1 in HGC27 and AGS cells transfected with the DDR1-expressing or control vector. **(d)** DDR1 overexpression induced actin cytoskeleton reorganization in GC cells. Quantifications of the relative fluorescence intensities of F-actin labeled with phalloidin staining in HGC27 and AGS cells transduced with the DDR1-expressing or control vector. All data were interpreted as means ± SEM from three independent experiments. The *P* values in panels (a), (b), (c) were calculated using Student’s *t*-test. ****P* < 0.001.

**
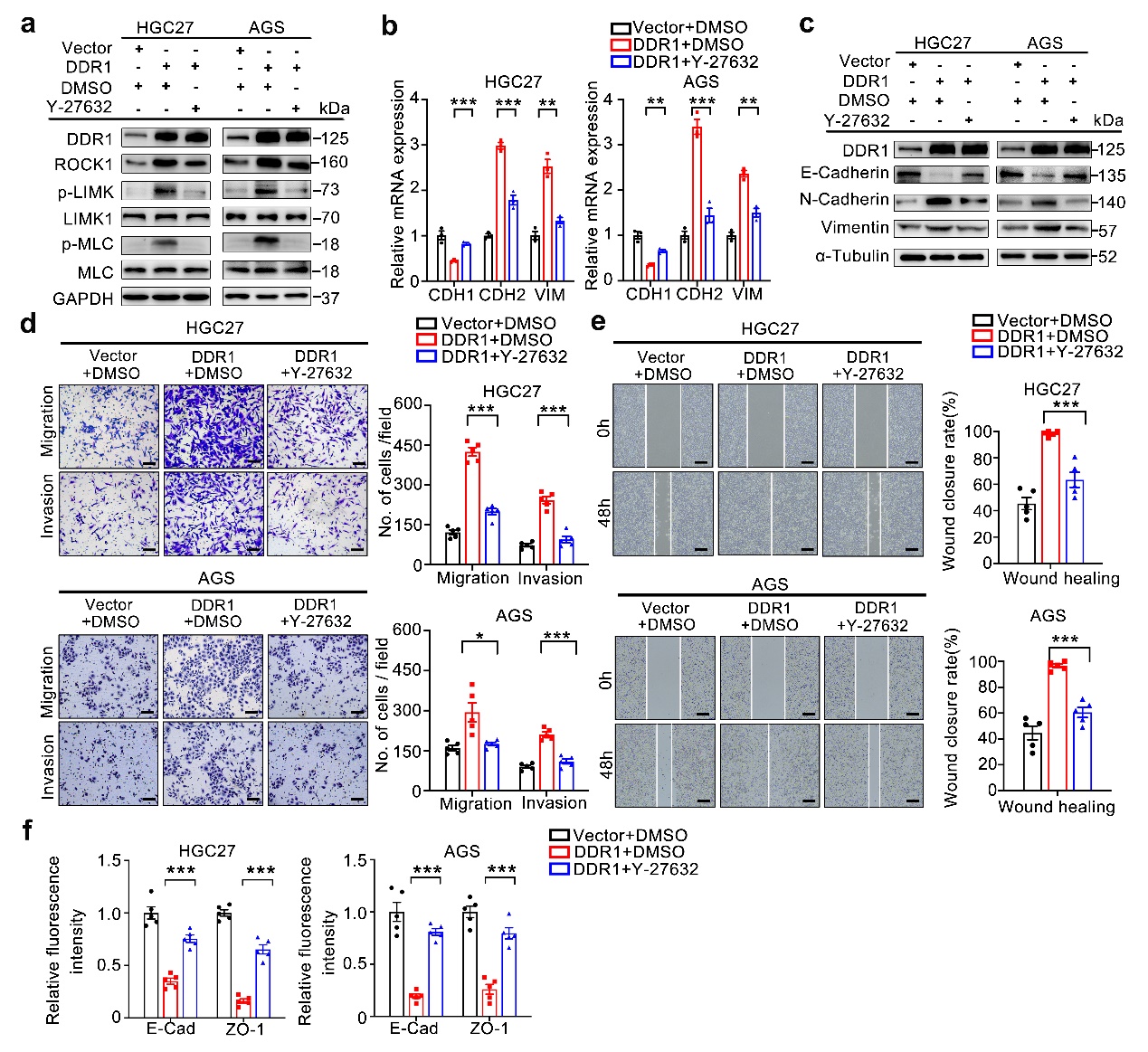
Figure S7. ROCK1 inhibition blocks DDR1-induced EMT and metastasis in GC cells *in vitro*.**

**(a)** Y-27632 suppressed DDR1-induced ROCK and its signaling pathway molecules. Western blot analysis was used to measure ROCK1, p-LIMK, LIMK1, p-MLC and MLC levels in DDR1-overexpressed HGC27 and AGS cells after Y-27632 treatment for 16 hours. **(b, c)** Y-27632 treatment decreased the EMT process in GC cells overexpressing DDR1. The mRNA and protein levels of EMT markers (E-cadherin, N-cadherin, and Vimentin) were evaluated using RT-PCR (b) and western blotting (c), respectively, in HGC27 and AGS cells transfected with DDR1-expressing or control vector following treatment with Y-27632. **(d)** Treatment with Y-27632 reduced the migration and invasion of DDR1-overexpressing GC cells. Transwell migration and Matrigel invasion assays were performed on HGC27 and AGS cells transduced with DDR1-expressing or control vector after 16 hours of Y-27632 treatment, and statistical analysis and representative images were obtained. Scale bar: 100μm. **(e)** The wound healing assays suggested that Y-27632 treatment decreased the motility of DDR1-overexpressing GC cells. Scale bar: 200 μm. **(f)** ROCK1 inhibition suppressed the downregulation of E-cadherin and ZO-1 in DDR1-overexpressed GC cells. The fluorescence intensities of E-cadherin and ZO-1 were quantified after treatment with Y-27632 (10uM) in HGC27 and AGS cells transfected with indicated vectors. All data were interpreted as means ± SEM from three independent experiments. The *P* values in panels (b), (d), (e), (f) were calculated using one-way ANOVA. **P* < 0.05, ***P* < 0.01, ****P* < 0.001.

**
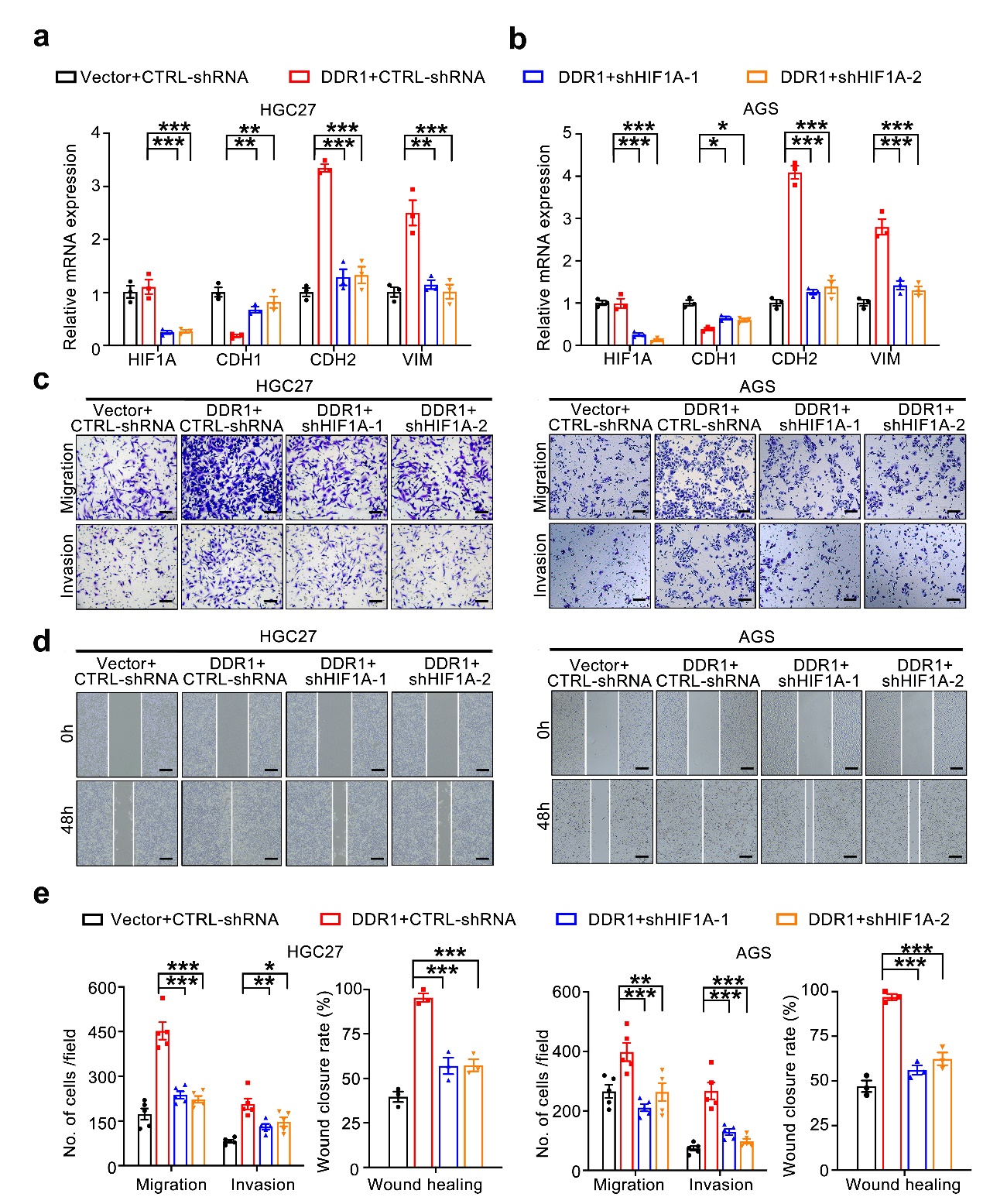
**

**Figure S8. HIF-1α is necessary for DDR1-incuced metastasis of GC cells *in vitro*.**

**(a, b)** *HIF1A* silencing suppressed the DDR1-induced EMT process in GC cells. The mRNA levels of E-cadherin, N-cadherin and Vimentin were analyzed by RT-PCR in HGC27 (a) and AGS (b) cells transfected with the indicated vectors. **(c)** *HIF1A* knockdown inhibited the migration and invasion in DDR1-overexpressed GC cells. Representative images of Transwell migration and Matrigel invasion assays of HGC27 and AGS cells transduced with the indicated vectors. Scale bar: 100μm. **(d)** The wound healing assay showed that *HIF1A* silencing decreased the motilities of DDR1-overexpressed GC cells. Scale bar: 200μm. **(e)** Statistical analysis of migration, Matrigel invasion and wound healing assays for HGC27 (left panel) and AGS (right panel) cells transduced with indicated vectors. All data were interpreted as means ± SEM from three independent experiments. The *P* values in panels (a), (b), (e) were calculated using one-way ANOVA. **P* < 0.05, ***P* < 0.01, ****P* < 0.001.


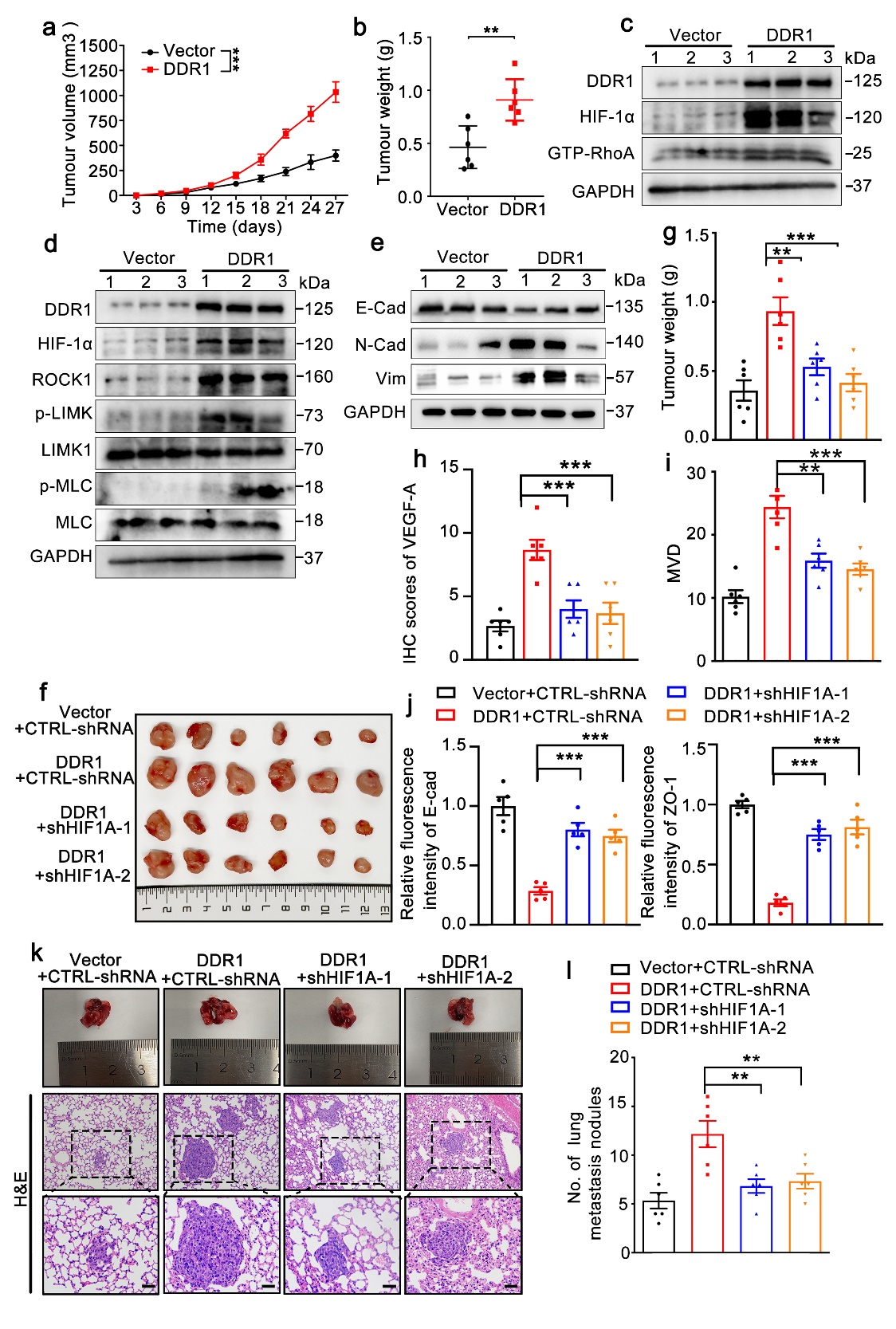


**. Figure S9. HIF-1α silencing retards DDR1-induced angiogenesis and metastasis *in vivo*.**

**(a)** The growth curve of subcutaneous tumours established by AGS-DDR1 or AGS-Vector cells demonstrated that DDR1 overexpression promoted tumour growth *in vivo* (n=6 per group). **(b)** Statistical analysis of subcutaneous tumour weights established by AGS-DDR1 or AGS-Vector cells. **(c, d)** DDR1 promoted RhoA/ROCK1 signaling in subcutaneous GC tumours. The protein levels of RhoA (c) and ROCK1 expression and the phosphorylation of LIMK and MLC (d) in tumours established by AGS-DDR1 cells or AGS-Vector cells were measured by western-blots. **(e)** DDR1 promoted the EMT processes in subcutaneous GC tumours. The protein levels of EMT markers (E-cadherin, N-cadherin and Vimentin) in tumours established by AGS-DDR1 cells or AGS-Vector cells were measured. **(f, g)** Silencing of *HIF1A* decreased the subcutaneous tumour weights established by AGS-DDR1 cells. Representative images (f) and quantifications of tumour weights (g) established by AGS cells transduced with indicated vectors. **(h)** *HIF1A* knockdown inhibited VEGF-A expression in tumours formed by AGS-DDR1 cells. IHC staining was performed to measure VEGF-A expression in the subcutaneous tumours from the 4 groups as indicated. **(i)** *HIF1A* knockdown decreased the microvessel densities (MVD) in DDR1-overexpressed tumours. Quantifications of MVD as indicated by CD31 expression in the subcutaneous tumours measured by IF staining. **(j)** *HIF1A* knockdown attenuated the downregulation of E-cadherin and ZO-1 in DDR1-overexpressed tumours. The expression of E-Cadherin and ZO-1 in the subcutaneous tumours were measured by IF staining. **(k, l)** Knockdown of *HIF1A* retarded the increase of lung metastases caused by DDR1-overexpression in mouse models. Representative images (k) and quantifications (l) of lung metastases established by tail vein injections measured by H&E staining. Scale bar: 25μm. All data are presented as the mean ± SEM from three independent experiments. The *P* values in panel (b) was calculated by Student’s *t*-test. The *P* values in panels (g-j) and (l) were calculated by one-way ANOVA. The *P* values in panel (a) was calculated by two-way ANOVA. **P* < 0.05, ***P* < 0.01, ****P* < 0.001.

**
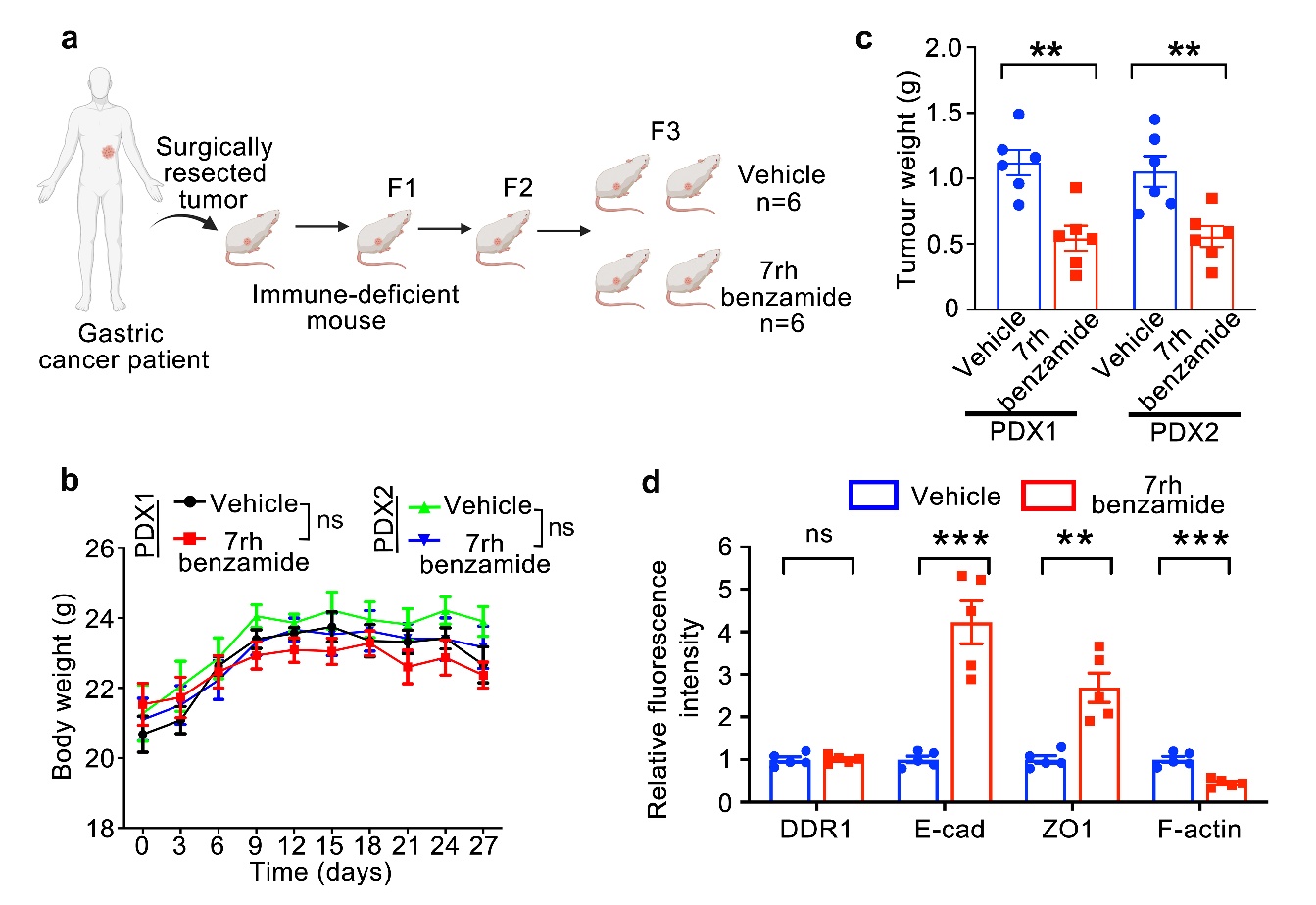
**

**Figure S10. Pharmacological inhibition of DDR1 significantly suppresses GC angiogenesis and metastasis in PDX and organoid models.**

**(a)** Schematic illustrations of the PDX model establishment derived from surgically resected GC tissues. **(b)** Nude mice weights of PDX models were recorded for each group (n=6). **(c)** The tumor weights of PDX models treated with a selective DDR1 inhibitor, 7rh benzamide, or vehicle were quantitatively analyzed (n=6 in each group). **(d)** The fluorescence intensity of DDR1, E-Cadherin, ZO-1, and F-actin in GC-derived organoids treated with 7rh benzamide or vehicle were statistically analyzed. All data were interpreted as means ± SEM from three independent experiments. The *P* values in panels (b) were calculated using one-way ANOVA. The *P* values in panels (c), (d) were calculated using Student’s *t*-test. ***P* < 0.01, ****P* < 0.001, ns: not significant.

**Supplementary Tables**

**Table S1. Baseline characteristics of patients according to the DDR1 expression.**

|  | | Low-DDR1  (n=89) | | High-DDR1  (n=93) | | *P* |
| --- | --- | --- | --- | --- | --- | --- |
| Age (years) | |  | |  | | 0.392 |
| <=55 | | 29 | | 37 | |  |
| >55 | | 60 | | 56 | |  |
| Gender | |  | |  | | 0.244 |
| Male | | 61 | | 55 | |  |
| Female | | 28 | | 38 | |  |
| pT categories | |  | |  | | 0.062 |
| T_1+2_ | | 23 | | 13 | |  |
| T_3+4_ | | 66 | | 80 | |  |
| pN categories | |  | |  | | 0.341 |
| N_0_ | | 31 | | 26 | |  |
| N_1+2+3_ | | 58 | | 67 | |  |
| Distant metastasis | |  | |  | | 0.668 |
| M_0_ | | 74 | | 74 | |  |
| M_1_ | | 15 | | 19 | |  |
| Borrmann type | |  | |  | | 0.992 |
| I-II | | 24 | | 24 | |  |
| III-IV | | 65 | | 69 | |  |
| E-cadherin | |  | |  | | 0.039 |
| Low | | 41 | | 58 | |  |
| High | | 48 | | 35 | |  |
| Vimentin | |  | |  | | 0.003 |
| Low | | 50 | | 31 | |  |
| High | | 39 | | 62 | |  |
| HIF-1α |  | |  | | 0.003 | |
| Low | 48 | | 29 | |  | |
| High | 41 | | 64 | |  | |

**Table S2. Univariate and multivariate analyses by Cox regressions according to overall survival for GC.**

| Variables | Univariate analysis | |  | Multivariable analysis | |  |
| --- | --- | --- | --- | --- | --- | --- |
|  | HR (95%CI) | *P* |  | HR (95% CI) | *P* | |
| Gender (Male *vs.* Female) | 0.65 (0.43-0.99) | 0.046 |  | ﻿1.01 (﻿0.82 to 1.25) | 0.926 | |
| Age | 1.01 (0.99-1.03) | 0.276 |  |  |  | |
| pT category (T_3-4_ vs. T_1-2_) | 10.64 (3.36-33.66) | <0.001 |  | ﻿5.39 (﻿1.67-17.43) | ﻿0.005 | |
| pN category (N_1-3_ vs. N_0_) | 8.85 (4.08 -19.19) | <0.001 |  | ﻿4.25 (﻿2.07-8.72) | <0.001 | |
| Distant metastasis (M_1_ vs. M_0_) | 5.69 (3.63-8.91) | <0.001 |  | ﻿3.40 (﻿2.15-5.35) | <0.001 | |
| CDH1 (High vs. Low) | 0.76 (0.50-1.15) | 0.195 |  |  |  | |
| Vimentin (High vs. Low) | 1.44 (0.94-2.21) | 0.091 |  |  |  | |
| HIF-1α (High vs. Low) | 1.37 (0.89-2.10) | 0.153 |  |  |  | |
| DDR1 (High vs. Low) | 1.53 (1.23-1.89) | <0.001 |  | ﻿1.55 (﻿1.25-1.92) | <0.001 | |

**Table S3. Univariate and multivariate analyses by Cox regressions according to recurrence-free survival for GC.**

| Variables | Univariable analysis | |  | Multivariable analysis | |
| --- | --- | --- | --- | --- | --- |
|  | HR (95%CI) | P |  | HR (95%CI) | P |
| Gender (Male vs. Female) | 0.67 (0.45-1.01) | 0.055 |  | ﻿0.96(﻿ 0.62-1.49) | 0.853 |
| Age | 1.01 (0.99-1.03) | 0.190 |  |  |  |
| pT category (T_3-4_ vs. T_1-2_) | 11.29 (3.57-35.07) | <0.001 |  | ﻿3.87(﻿1.19-12.63) | ﻿0.025 |
| pN category (N_1-3_ vs. N_0_) | 7.04 (3.53-14.03) | <0.001 |  | ﻿ 5.10(﻿2.27-11.42) | <0.001 |
| Distant metastasis (M_1_ vs. M_0_) | 5.89 (3.75-9.25) | <0.001 |  | ﻿3.03(﻿1.84-5.00) | <0.001 |
| VEGF-A (High vs. Low) | 1.26 (1.01-1.58) | 0.045 |  | ﻿ 0.95(﻿0.58-1.56) | ﻿0.839 |
| CDH1 (High vs. Low) | 0.88 (0.72-1.08) | 0.227 |  |  |  |
| VIM (High vs. Low) | 1.18 (0.96-1.45) | 0.122 |  |  |  |
| HIF1A (High vs. Low) | 1.16 (0.94-1.43) | 0.174 |  |  |  |
| DDR1 (High vs. Low) | 1.53(1.23-1.89) | <0.001 |  | ﻿2.58(﻿﻿1.57-4.25) | <0.001 |

**Table S4. Antibodies used for immunohistochemistry.**

| Antibody | Provider | Dilution |
| --- | --- | --- |
| DDR1 | Cell signaling Technology, #5583 | 1:100 |
| VEGF-A | Abcam, #ab1316 | 1:100 |
| HIF-1α | Abcam, #ab51608 | 1:50 |
| E-cadherin | Abcam, #ab40772 | 1:250 |
| Vimentin | Cell signaling Technology, #5741 | 1:100 |
| CD31 | Abcam, #ab182981 | 1:300 |

**Table S5. Antibodies used for immunoblotting.**

| Antibody |  | Provider | Dilution |
| --- | --- | --- | --- |
| DDR1 |  | Cell signaling Technology, #5583 | 1:1000 |
| VEGF-A |  | Abcam, # ab46154 | 1:1000 |
| HIF-1α |  | Abcam, #ab51608 | 1:500 |
| E-cadherin |  | Cell signaling Technology, #3195 | 1:1000 |
| Vimentin |  | Cell signaling Technology, #5741 | 1:1000 |
| N-cadherin |  | Cell signaling Technology, #13116 | 1:1000 |
| ROCK1 |  | Cell signaling Technology, #4035 | 1:1000 |
| phospho-LIMK1 Thr^508^ |  | Abcam, #ab38508 | 1:1000 |
| LIMK1 |  | Cell signaling Technology, #3842S | 1:1000 |
| p-MLC Ser^19^ |  | Cell signaling Technology, #3675 | 1:1000 |
| MLC |  | Cell signaling Technology, #8505 | 1:1000 |
| HA |  | Millipore, #05-904 | 1:1000 |
| Flag  FGF2  PDGFB |  | Cell signaling Technology, #8146S  Abclonal, #A11488  Abclonal, #A22035 | 1:1000  1:1000  1:1000 |

**Table S6. Antibodies used for immunoprecipitation.**

| Antibody | Provider | Dilution |
| --- | --- | --- |
| DDR1 | Cell signaling Technology, #5583 | 1:50 |
| HIF-1α | Abcam, #ab51608 | 1:50 |
| HA | Merck, #05-904 | 1:50 |
| Flag | Cell signaling Technology, #8146 | 1:50 |

**Table S7. Antibodies used for immunofluorescence.**

| Antibody | Provider | Dilution |
| --- | --- | --- |
| CD31 | Cell signaling Technology, #3528 | 1:100 |
| HIF-1α | Abcam, #ab51608 | 1:100 |
| Ubiquitin | Abcam, #ab7780 | 1:200 |
| DDR1 | Cell signaling Technology, #5583S | 1:1000 |
| E-cadherin | Abcam, #ab40772 | 1:250 |
| ZO-1 | Abcam, #ab190085 | 1:250 |

**Table S8. Primers sequences for qRT-PCR.**

| Gene name | Primer sequences |
| --- | --- |
| DDR1 | F：5’-ATGGAGCAACCACAGCTTCTC-3’ |
|  | R：5’-CTCAGCCGGTCAAACTCAAACT-3’ |
| GAPDH | F：5’-ACAACTTTGGTATCGTGGAAGG-3’ |
|  | R：5’-GCCATCACGCCACAGTTTC-3’ |
| VEGF-A | F：5’-TTGCCTTGCTGCTCTACCTCCA -3’ |
|  | R：5’-GATGGCAGTAGCTGCGCTGATA-3’ |
| VEGF-B | F：5’-AAGGACAGTGCTGTGAAGCCAG-3’ |
|  | R：5’-TGGAGTGGGATGGGTGATGTCA -3’ |
| VEGF-C | F：5’-GCCAATCACACTTCCTGCCGAT-3’ |
|  | R：5’-AGGTCTTGTTCGCTGCCTGACA-3’ |
| HIF-1α | F：5’-GAACGTCGAAAAGAAAAGTCTCG-3’ |
|  | R：5’- CCTTATCAAGATGCGAACTCACA-3’ |
| CDH1 | F：5’-CTTTGACGCCGAGAGCTACA -3’ |
|  | R：5’-TTTGAATCGGGTGTCGAGGG -3’ |
| CDH2 | F：5’-CGTCTGTAGAGGCTTCTGGTG -3’ |
|  | R：5’-AATCTGCAGGCTCACTGCTC-3’ |
| Vimentin | F：5’-GTTGACAATGCGTCTCTGGC-3’ |
|  | R：5’-CGTGAGGTCAGGCTTGGAAA-3’ |
| GLUT1 | F：5’-TTGGCTCCCTGCAGTTTGGC-3’ |
|  | R：5’-CCCCATAGCGGTGGACCCAT-3’ |
| ALDOA | F：5’-GCTGTCACTGGGATCACCTTC-3’ |
|  | R：5’-GCTCGGAGTGTACTTTCCTTGA-3’ |
| PGK1 | F：5’-CCACTGTGGCTTCTGGCATA-3’ |
|  | R：5’-ATGAGAGCTTTGGTTCCCCG-3’ |
| TFRC | F：5’-ACCATTGTCATATACCCGGTTCA-3’ |
|  | R：5’-CAATAGCCCAAGTAGCCAATCAT-3’ |
|  |  |
